# Comparative Transcriptomic Analysis of Key Genes Involved in Citrinin Biosynthesis in *Monascus purpureus*

**DOI:** 10.3390/jof9020200

**Published:** 2023-02-03

**Authors:** Yingying Huang, Chenglong Yang, István Molnár, Shen Chen

**Affiliations:** 1 Institute of Agricultural Engineering Technology, Fujian Academy of Agricultural Sciences, Fuzhou 350003, China; 2 Key Laboratory of Subtropical Characteristic Fruits, Vegetables and Edible Fungi Processing (Co-Construction by Ministry and Province), Ministry of Agriculture and Rural Affairs, Fuzhou 350003, China; 3 Fujian Key Laboratory of Agricultural Products (Food) Processing, Fuzhou 350003, China; 4 VTT Technical Research Centre of Finland, 02100 Espoo, Finland

**Keywords:** *Monascus purpureus*, secondary metabolites, pigment, citrinin, RNA-seq analysis

## Abstract

*Monascus* pigments (MPs) display many beneficial biological activities and have been widely utilized as natural food-grade colorants in the food processing industry. The presence of the mycotoxin citrinin (CIT) seriously restricts the application of MPs, but the gene regulation mechanisms governing CIT biosynthesis remain unclear. We performed a RNA-Seq-based comparative transcriptomic analysis of representative high MPs-producing *Monascus purpureus* strains with extremely high vs. low CIT yields. In addition, we performed qRT-PCR to detect the expression of genes related to CIT biosynthesis, confirming the reliability of the RNA-Seq data. The results revealed that there were 2518 differentially expressed genes (DEGs; 1141 downregulated and 1377 upregulated in the low CIT producer strain). Many upregulated DEGs were associated with energy metabolism and carbohydrate metabolism, with these changes potentially making more biosynthetic precursors available for MPs biosynthesis. Several potentially interesting genes that encode transcription factors were also identified amongst the DEGs. The transcriptomic results also showed that *citB, citD, citE, citC* and perhaps *MpigI* were key candidate genes to limit CIT biosynthesis. Our studies provide useful information on metabolic adaptations to MPs and CIT biosynthesis in *M. purpureus*, and provide targets for the fermentation industry towards the engineering of safer MPs production.

## 1. Introduction

The filamentous fungus *Monascus* spp. has a long history of application as a traditional medicine, and it is commonly used to produce various traditional oriental foods such as rice wine, tofu curds, and fermented fish [1]. A variety of beneficial secondary metabolites are synthesized by *Monascus,* such as the *Monascus* pigments (MPs), monacolins (HMG-CoA reductase inhibitors), γ-amino butyric acid (GABA; hypotensive agent), and other bioactive components [2]. Therefore, *Monascus* fermentation products represent enormous commercial value, and offer a great potential for the development of therapeutic agents due to their antioxidant, anti-atherosclerotic, and antimicrobial properties [3,4]. MPs are widely utilized as natural and safe food colorants, condiments, and preservatives in meat (sausages and hams) and fish products to dye food and prolong the shelf life. However, the mycotoxin citrinin (CIT) may also be produced by *Monascus* strains simultaneously with MPs, compromising safety and seriously restricting the application of MPs in the food industry [5]. CIT displays mutagenic, carcinogenic, nephrotoxic, teratogenic, and hepatotoxic activities in mammals. Accordingly, limits of CIT in MPs products have been legislated as one of the important global food safety indicators. The maximum tolerance limit of CIT in red yeast rice (RYR, a rice-based fermentation product, also known as *Monascus*-fermented rice) is 2000, 200, and 50 ppb (2, 0.2, and 0.05 mg/kg) in the European Union (UC Commission Regulation No.212/2014.), Japan, and Korea, respectively. Compared to other countries, the Chinese National Standard GB1886.181–2016 stipulates a more stringent critical upper limit on the allowable CIT content in *Monascus* RYR with 0.04 mg/kg (calculated for 500 U/g of MPs). However, the contents of CIT in the high color value MPs products in China generally exceed the above standards, ranging from 0.23 to 20.65 mg/kg [6]. This hinders the export of MP products and the development of the MPs industry.

Many methods have been investigated to enhance the production efficiency of MPs and control CIT production. The traditional strategy is to optimize the fermentation process parameters, including the adjustment of the media composition, culture conditions, and downstream procedures in order to reduce CIT biosynthesis, or to degrade and remove CIT [7,8]. However, maintaining or increasing the MPs yield while reducing the CIT production by changing the fermentation parameters does not block CIT biosynthesis completely. Thus, the identification of CIT low- or non-producing *Monascus* strains is highly important to control CIT production. Such strains have traditionally been generated by physical or chemical random mutagenesis and genetic recombination. However, such methods are relatively inefficient; the nature of the generated mutations is mostly unknown; beneficial mutations may remain unstable due to gene repair; and mutations compromising the growth and MPs productivity may accumulate [9,10]. Therefore, investigating the mechanisms of the global regulation of CIT biosynthesis and the genetic engineering of these processes may be considered the core technology to realize the elimination of CIT from MPs products [11].

The carbon skeletons of CIT and MPs are derived from the polyketide and the fatty acid biosynthesis pathways. These biosynthetic routes utilize the common precursors acetyl-CoA and malonyl-CoA [6,7,8,9]. These common precursors are converted by recursive Claisen condensations to a pentaketide (CIT) or a hexaketide (MPs) precursor by separate, pathway-specific polyketide synthase (PKS) enzymes, and to β-ketooctanoic or β-ketodecanoic acid moieties by a dedicated fatty acid synthase (FAS) for MPs assembly. Formation of the mature CIT and MPs products requires a series of complex chemical steps, catalyzed by enzymes encoded in the separate CIT and MPs biosynthetic gene clusters (BGCs) that are controlled by different cluster-specific regulators. However, many aspects of the higher-level regulation of CIT and MPs biosynthesis remain unknown or controversial.

CIT is typically produced in conjunction with MPs in *M. purpureus*, and the optimization of the fermentation process for increased MPs production is often accompanied by an unwanted rise in CIT production. Importantly, comparative transcriptomic analyses have been applied to investigate gene transcription in this fungus under different fermentation conditions (such as different carbon/nitrogen sources, special additives, light treatment, etc.) [12,13,14,15]. These studies often concentrated on the biosynthesis of MPs, although several reports also investigated the expression of the CIT pathway in different *M. purpureus* strains and fermentation conditions. Some of these reports also suggested strategies to reduce CIT production. Thus, supplementation of inorganic nitrogen to the fermentation media resulted in reductions in the mRNA levels of *ctnA* and *pksCT*, while adding genistein down-regulated the expression of *orf5, pksCT, orf3, orf1, orf6*, and *ctnE* [16,17,18]. However, the expression level of *pksCT* was low both in *M. purpureus* YY-1 and its random mutant with low CIT production [19]. We assumed that CIT formation may be prevented during MPs production by modulating the expression of key genes whose protein products support, participate in, or regulate CIT biosynthesis. Reduction of CIT production would then also be expected to improve the metabolic flux of the precursors towards the biosynthesis of MPs. Considering that the regulation of CIT biosynthesis and its integration into the higher-level genetic circuits of *M. purpureus* is largely unknown, we decided to compare the transcriptomic profiles of different strains of *M. purpureus* that produce various amounts of CIT due to their unknown genetic differences. Hence, the aim of this study was to identify the potential key genes regulating the partitioning of metabolic resources between MPs and CIT biosynthesis in the fungal cells. Thus, we conducted a comparative transcriptomic analysis between two representative high MPs-producing *M. purpureus* strains that differ in their CIT production: one with extremely high and another with low CIT yield. We utilized high-throughput RNA-seq validated by qRT-PCR, identifying genes with differential expressions at the peak period of secondary metabolite biosynthesis.

## 2. Materials and Methods

### 2.1. Strain and Culture Methods

*Monascus* strains were isolated from RYR collected from various manufacturers and local markets in China, and preserved in our laboratory. Detailed information about these strains has been described previously [20]. *Monascus* strains were inoculated onto slant cultures (MEA: 6% malt extract, 1.5% agar) at 35 °C for 10 d for initial growth.

The seed and liquid culture medium consisted of 6% glucose, 2% peptone, 0.15% NaNO_3_, 0.05% KH_2_PO_4_, 0.05% MgSO_4_·7H_2_O, 0.15% K_2_HPO_4_, MnSO_4_·H_2_O, and 0.01% ZnSO_4_·H_2_O in distilled water without pH adjustment, sterilized at 121 °C for 25 min.

Seed cultivation: The spore suspension of each strain was harvested after 10 days of growth by flooding the slant culture with sterilized water, and the concentration was adjusted to approximately 2 × 10^7^ spores per mL with sterile distilled water. Next, 5 mL of spore suspension was inoculated into a 250 mL Erlenmeyer flask containing 50 mL of a seed culture medium. The resulting culture was cultivated at 35 °C with shaking at 180 rpm for 2 days.

Liquid production of MPs and CIT: The seed liquid culture was subsequently transferred into a 1 L Erlenmeyer flask with 200 mL of a liquid fermentation medium at a ratio of 15% (*v/v*) and cultured at 35 °C with shaking at 180 rpm for 160 h.

Solid-state production of MPs and CIT [21]: Long-grained non-glutinous rice (50 g) was used as the substrate in a 500 mL Erlenmeyer flask. The rice grains were washed and soaked under water overnight at room temperature, then the steeped rice was autoclaved at 121 °C for 30 min. After cooling to room temperature, the steamed rice was inoculated and mixed evenly with 10% (*v/w*) of the seed culture, and cultivated at 35 °C in a constant temperature incubator for 13 d. During this period, the sterilized water was replenished regularly to maintain the moisture content. After cultivation, the resulting RYR was dried at 60 °C for 6 h and then ground into a powder.

### 2.2. Quantitative Analysis and Strain Identification

The mycelium was harvested by filtering culture broths, washed with distilled water, then dried at 60 °C. The total biomass was determined as the dry mycelium weight and expressed as dry biomass weight (g)/fermentation broth (100 mL). MPs and CIT were determined following the Chinese National Standard GB1886.19-2015 and GB 5009.222-2016, respectively. The liquid fermentation broth of each flask (1 mL) or the powder of red yeast rice (0.1 g) was extracted with 25 mL of 70% ethanol at 60 °C for 1 h. After filtration and cooling to room temperature, the extracted liquid or the filtrate was serially diluted and the optical density (OD) was measured using an ultraviolet-visible spectrophotometer (UV-240, Shimadzu, Japan) against a 70% ethanol blank at 505 nm. The yield of MPs was expressed as optical density (OD) units per mL or gram (U/mL or U/g) multiplied by the dilution factor. Subsequently, the supernatant liquid was filtered with a 0.45 μm filter, and the CIT content was analyzed by high performance liquid chromatography/tandem mass spectrometry (HPLC/MS/MS) on a SHIMADZU 2010-API 4000 QTRAP using an RF-10 Axl fluorescence detector (λex = 331 nm, λem = 500 nm) and a Phenomenex Synergi Fusion-RP 80A C_18_ column (4 μm, 2.0 × 150 mm). Elution was performed at 38 °C with 0.2% acetic acid/acetonitrile of 45:55 (*v/v*, pH 2.5) and a run time of 9 min at a flow rate of 0.4 mL/min. Commercial CIT (Sigma, St. Louis, MO, USA) was used as the standard.

Seventy *Monascus* strains preserved in our laboratories were taxonomically characterized by combining ITS sequence analysis with the determination of key physiological and biochemical characteristics. The hydrolyzed gelatin test employed a sterilized culture medium consisting of 0.5% peptone and 15% gelatin, with the pH ranging from 7.0 to 7.2. *Monascus* spores were inoculated in the center of the gelatin media-containing test tubes by stabbing. Together with two un-inoculated tubes as the blank control, all cultures were incubated at 30 °C for 7 days. Because the gelatin culture medium would melt above 20 °C, the test tubes were cooled in a refrigerator at 4 °C until the control tubes solidified completely. The tubes were subsequently kept under 20 °C until recording the results. The hydrolyzed gelatin test was recorded as positive when part or all the gelatin medium remained liquid [15,22]. *Monascus* strains were identified by nuclear ribosomal RNA internal transcribed spacer (ITS) sequence analysis. The genomic DNA of each strain was extracted using the OMEGA Soil DNA Kit (Omega Bio-Tek, Norcross, GA, USA). The ITS1-5.8S rRNA gene intergenic region of each strain was amplified with the forward primer ITS-1 (5′-TCCGTAGGTGAACCTGCGG-3′) and the reverse primer ITS-4 (5′-TCCTCCGCTTATTGATATGC-3′), as described in our previous studies [22]. The PCR reaction contained 2 μL of DNA template, 10 μL of 2X Taq Master Mix, 10 μL of ddH_2_O, and 1 μL of each primer ITS1 and ITS4. Thermal cycling consisted of initial denaturation at 94 °C for 5 min, followed by 30 cycles consisting of denaturation at 94 °C for 1 min, annealing at 55 °C for 1 min, and extension at 72 °C for 1 min, with a final extension of 10 min at 72 °C. Purified PCR products were sequenced using an ABI prism 3730 DNA analyzer (Applied Biosystems, Waltham, MA, USA), and the DNA sequences were compared with Blast searches against the NCBI GenBank database.

### 2.3. Genome Sequencing and Assembly

DNA for genome sequencing was extracted with the SDS method [23]. The harvested DNA was analyzed by agarose gel electrophoresis and quantified by a Qubit^®^ 2.0 Fluorometer (Thermo Scientific, Waltham, MA, USA). A total amount of 1 µg DNA per sample was used for the sequencing library preparations. Sequencing libraries were generated using NEBNext^®^ Ultra^TM^ DNA Library Prep Kit for Illumina (NEB, Ipswich, MA, USA) following the recommendations of the manufacturer, and index codes were added to attribute sequences to each sample. The genomes of *Monascus* strains M3 and M34 were sequenced using Illumina NovaSeq PE150 at the Beijing Allwegene Technology Co., Ltd. Illumina PCR adapters and low-quality sequences were filtered from the paired-end reads. The good quality paired reads were assembled using SOAP de novo [24,25] (http://soap.genomics.org.cn/soapdenovo.html (accessed on 1 October 2021)), SPAdes [26] (http://cab.spbu.ru/software/spades/ (accessed on 1 October 2021)) and ABySS [27] (http://www.bcgsc.ca/platform/bioinfo/software/abyss (accessed on 1 October 2021)) into scaffolds, and the filtered reads were used for gap-closing.

### 2.4. Transcriptome Sequencing and Differential Gene Expression Analysis

Total RNA samples from each strain were extracted from flash-frozen, ground mycelia (independent biological triplicates), using the mirVanaTM miRNA ISOlation Kit (Ambion-1561, Austin, TX, USA) with on-column treatment with DNase I (Ambion, to remove genomic DNA) following the standard protocol of the manufacturer. The extracted total RNA was quantified and checked for integrity using a NanoDrop 2000 spectrophotometer (Thermo Fisher Scientific, Inc., Wilmington, NC, USA) and an Agilent 2100 Bioanalyzer (Agilent Technologies, Santa Clara, CA, USA), respectively. Next, the total RNA for each sample cDNA library was sequenced on an Illumina HiSeq^TM^ 2500 sequencer platform using 150 paired-end sequencing (OE Biotech Co., Ltd., Shanghai, China) [28]. Raw reads were quality-filtered by removing low-quality reads, adapter sequences, and poly-N tracks using RseQC v.2.6.4. The ratio of the valid bases, Q30, and GC content of the clean data were calculated. The clean reads were mapped and compared to the reference genome sequence *M. purpureus* Monpu1 (JPI: https://mycocosm.jgi.doe.gov/Monru1/Monru1.home.html (accessed on 1 May 2021)) using HISAT2 software v.2.2.1.0. The normalized transcript abundance described as FPKM values (fragments per kilobase of transcript per million fragments mapped) were used to estimate the gene expression levels. To ensure data accuracy, the data was standardized by correcting for the sequencing depth and performing hypothesis test correction based on statistical analysis. The DESeq R package (v.1.18.0) was used to analyze differential gene expression based on the negative binomial distribution. Genes showing *p* < 0.05 and FC (fold change) >2 or <0.5 were set as the threshold for significantly different expressions.

### 2.5. Comparative Genomics Analysis and SNP Identification

SNPs (Single Nucleotide Polymorphisms) were identified by genome sequence alignment between M3 and M34 using MUMmer and LASTZ. For the transcriptomes, reads were mapped onto the reference genome (Monpu1) using the Burrows Wheeler Aligner (bwa) software [29], and SNPs were identified using GATK [30] with thresholds for mapping quality (>50) and read depth (>10); SNPs had to appear in forward and reverse reads. Functional annotation was performed for the identified SNPs to investigate their genomic location and variation information using the ANNOVAR software [31].

### 2.6. Functional Annotation

Gene function was annotated by the following frequently used databases: NR (NCBI non-redundant protein sequences), Swiss-Prot (a manually annotated and reviewed protein sequence database), KO (Kyoto encyclopedia of genes and genomes pathways ontology database), KOG/COG (Clusters of Orthologous Groups of proteins), Pfam (Protein family), TCDB (transporter classification database), eggNOG (evolutionary genealogy of genes: Nonsupervised Orthologous Groups) and GO (Gene Ontology), using BLAST (Basic Local Alignment Search Tool) with a cutoff E-value of 10^−5^. Secondary metabolite biosynthetic gene clusters were further analyzed by antiSMASH [32]. Carbohydrate-active enzymes (CAZy) were predicted by the Carbohydrate-Active enZYmesDatabase. The annotations of differentially expressed genes (DEGs) against the above databases and intersections were graphically represented with an UpSet plot. GO- and KEGG-enrichment analyses of DEGs were implemented using the cluster profile (v.3.28.1) R package based on Wallenius non-central hypergeometric distribution and the KOBAS software, respectively. GO terms with corrected *p* < 0.05 were considered significantly enriched with DEGs.

### 2.7. Reverse Transcription Quantitative Real-Time PCR Analysis of Key Genes in Citrinin Biosynthesis

Gene expression profiles were quantified by RT-qPCR, with a two-step reaction process: reverse transcription (RT) and quantitative PCR. The reverse transcription (RT) reaction was performed in a GeneAmp^®^ PCR System 9700 (Applied Biosystems, Waltham, MA, USA) as follows: 0.5 μg total RNA, 2 μL of 4× gDNA Wiper Mix, and nuclease-free water in a total volume of 10 μL was incubated at 42 °C for 2 min. After adding 2 μL of 5× HiScript II Q RT SuperMix II, the mixture was incubated at 37 °C for 15 min and 5 s at 85 °C. The 10 μL RT reaction mixture was then held at −20 °C.

The qPCR step was carried out using a LightCycler^®^ 480 II Real-time PCR Instrument (Roche, Switzerland), and reactions were incubated in 384-well optical plates (Roche, Switzerland) with a 10 μL PCR reaction mixture including 1 μL of cDNA, 5 μL of 2× ChamQ SYBR qPCR Master Mix (Vazyme, Nanjing, China), 0.2 μL of 10 μM forward primer, 0.2 μL of 10 μM reverse primer and 3.6 μL of nuclease-free water. The amplification program was as follows: denaturation at 94 °C for 30 s, followed by 45 cycles of denaturation at 94 °C for 5 s, then annealing and extension at 60 °C for 30 s. At the end of the PCR cycle, a melting curve analysis was performed to validate the specific generation of the expected PCR product. The relative expression level of each gene was normalized and calibrated by the 2^−ΔΔCt^ method, with the expression in M3 as the control, and with glyceraldehyde-3-phosphate dehydrogenase (GAPDH) as an endogenous control. The primers were designed by using the Primer Premier 5.0 software, based on the mRNA sequences obtained from the NCBI database. The primer sequences are listed in Table S1. Three biological replicates of each sample were analyzed by the RT-qPCR assay with three technical replicates for each of the selected genes.

### 2.8. Statistical Analysis

All the experiments were performed in independent biological triplicates. The results are expressed as the means ± standard deviation (SD) in the figures. The statistical significance was determined by one-way analysis of variance (ANOVA), with *p* < 0.05 indicating significant differences between different samples.

## 3. Results and Discussion

### 3.1. Screening and Verification of High MPs-Producing M. purpureus Strains

*M. purpureus* is the predominant species for the efficient production of MPs, but it also has a strong capacity for producing CIT [25]. To identify representative *M. purpureus* varieties with extremely high vs. low MPs or CIT yield, we selected 70 *Monascus* strains preserved in our laboratories for physiological and biochemical identification combined with the ITS sequences analysis. The results showed that 18 strains could grow on glucose, maltose, fructose, sucrose, and lactose, but cannot grow on sorbose. The hydrolyzed gelatin test for these strains was positive. Then, two pairs of primers (ITS1/ITS4) were used to amplify and sequence different fragments of the ITS1-5.8S rDNA-ITS2 gene and to identify their species. The amplified ITS1/ITS4 fragments of fourteen strains show the highest similarity with *M. purpureus* (Table S2). Based on these results, we concluded that these fourteen strains belong to *M. purpureus*.

**Figure 1:**
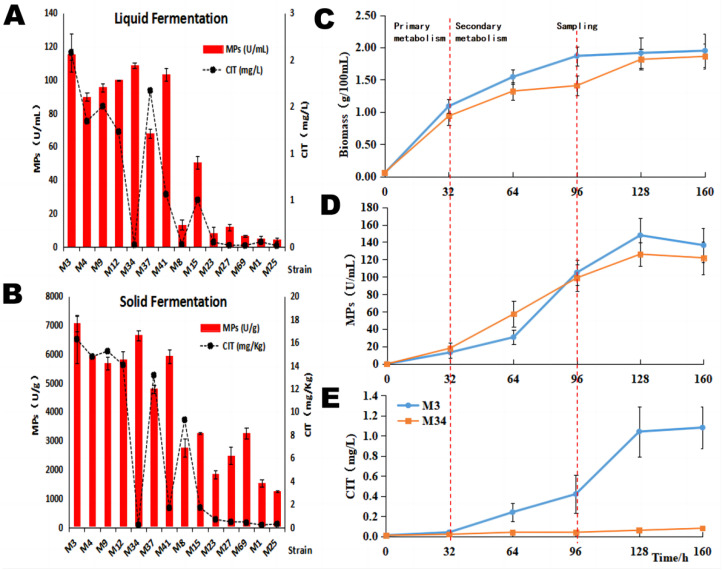
Overview of MPs and CIT production by *M. purpureus* strains. Shake flask fermentations in liquid media (**A**) and solid-state fermentations (**B**), using *M. purpureus* M3 and 13 additional strains. Time course analysis of *M. purpureus* M3 and M34 cell growth (**C**), relative yields of MPs (**D**), and relative productions of CIT (**E**). Results are shown as the mean of three biological replicates; the error bars represent the standard deviation of the means (n = 3).

Next, we conducted liquid-state fermentations with these strains, taking advantage of the short fermentation periods of *Monascus* under such conditions. After four days of cultivation, *M. purpureus* M3 showed the highest production of both MPs and CIT, 114.96 ± 3.32 U/mL and 2.08 ± 0.21 mg/L, respectively (Figure 1A). In contrast, *M. purpureus* M34 produced high amounts of MPs (108.87 ± 1.64 U/mL) that were not significantly different from that of strain M3 (*p* > 0.05). In contrast, strain M34 afforded approximately 104-fold less CIT (0.02 ± 0.01 mg/L) compared to that of strain M3. Considering that solid-state fermentation on cooked rice is the traditional method widely used in the industry to produce MPs with *Monascus* strains, all strains were also cultivated using this method (Figure 1B). Both *M. purpureus* M3 and M34 exhibited high MPs yields of 7050 ± 259.75 U/g and 6643 ± 167.47 U/g, respectively. The CIT yield of M3 was 16.28 ± 2.08 mg/kg, while M34 produced 0.21 ± 0.10 mg/kg of this mycotoxin. Thus, we chose M3 (the control group) and M34 (the experiment group) as the representatives for high MPs-producing *M. purpureus* strains with extremely high vs. low CIT yield, respectively.

### 3.2. Growth and Production Kinetics Analysis

To further investigate CIT metabolism in these two representative strains, the kinetics of growth and those of the biosynthesis of MPs and CIT were analyzed. The biomass amounts and the yields of MPs and CIT were determined every 32 h. The biomass amounts increased rapidly in the early logarithmic growth phase (0–32 h) and peaked at 1.7–2.2 g/100 mL in the stationary phase (Figure 1C). The production of MPs and CIT increased rapidly in the late logarithmic growth phase (64–128 h, Figure 1D,E). We found that the yields of MPs (126.10–147.82 U/mL) and the mycelial biomass were similar in the two strains, whereas the CIT yield of M34 (0.06 ± 0.52 mg/L) was significantly lower than that of M3 (1.04 ± 0.11 mg/L, *p* < 0.05) after 128 h of cultivation. The transcription of secondary metabolism-related genes was expected to be maximal during the rapid biosynthesis of MPs and CIT. Therefore, mycelia were collected at 96 h of the liquid-state cultivation, corresponding to the middle of the rapid increase stage of CIT and MPs production, to perform a comparative transcriptomic analysis.

### 3.3. Genome Sequencing and Annotation

**Figure 2:**
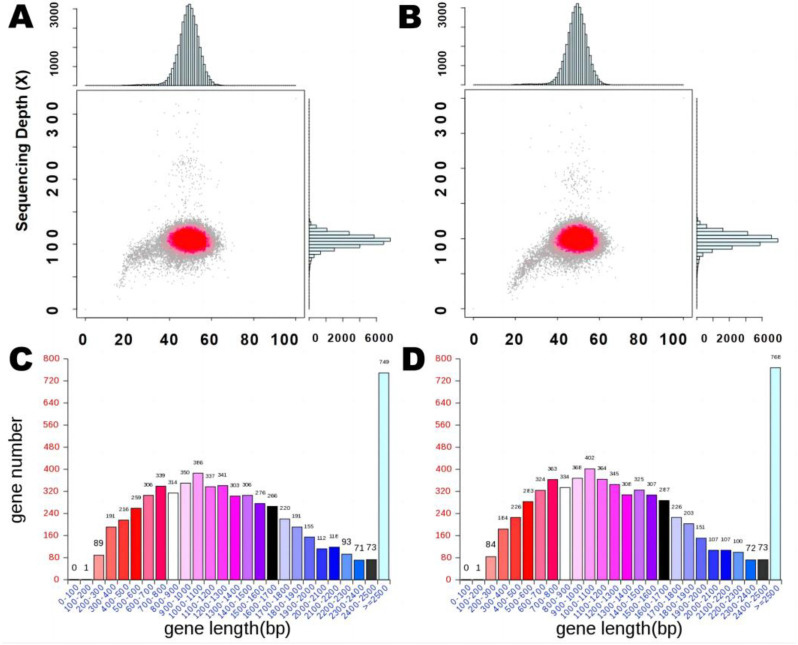
Genome sequence characteristics of *M. purpureus* M3 and M34. Correlation analysis between GC content and sequencing depth in (**A**) M3 and (**B**) M34; gene length distribution in (**C**) M3 and (**D**) M34.

To further characterize the genetic and evolutional similarity of these two fungi, we first sequenced and compared the genomes of the selected high MPs-producing *M. purpureus* strains M3 and M34. After quality control and assembly, genome sequences of nearly identical size and GC content were obtained (Table 1, Figure 2A,B). The numbers of predicted genes, the total gene lengths, the gene length distributions, and the numbers of tRNAs were also similar between these two strains (Table 2, Figure 2C,D).

**Figure 3:**
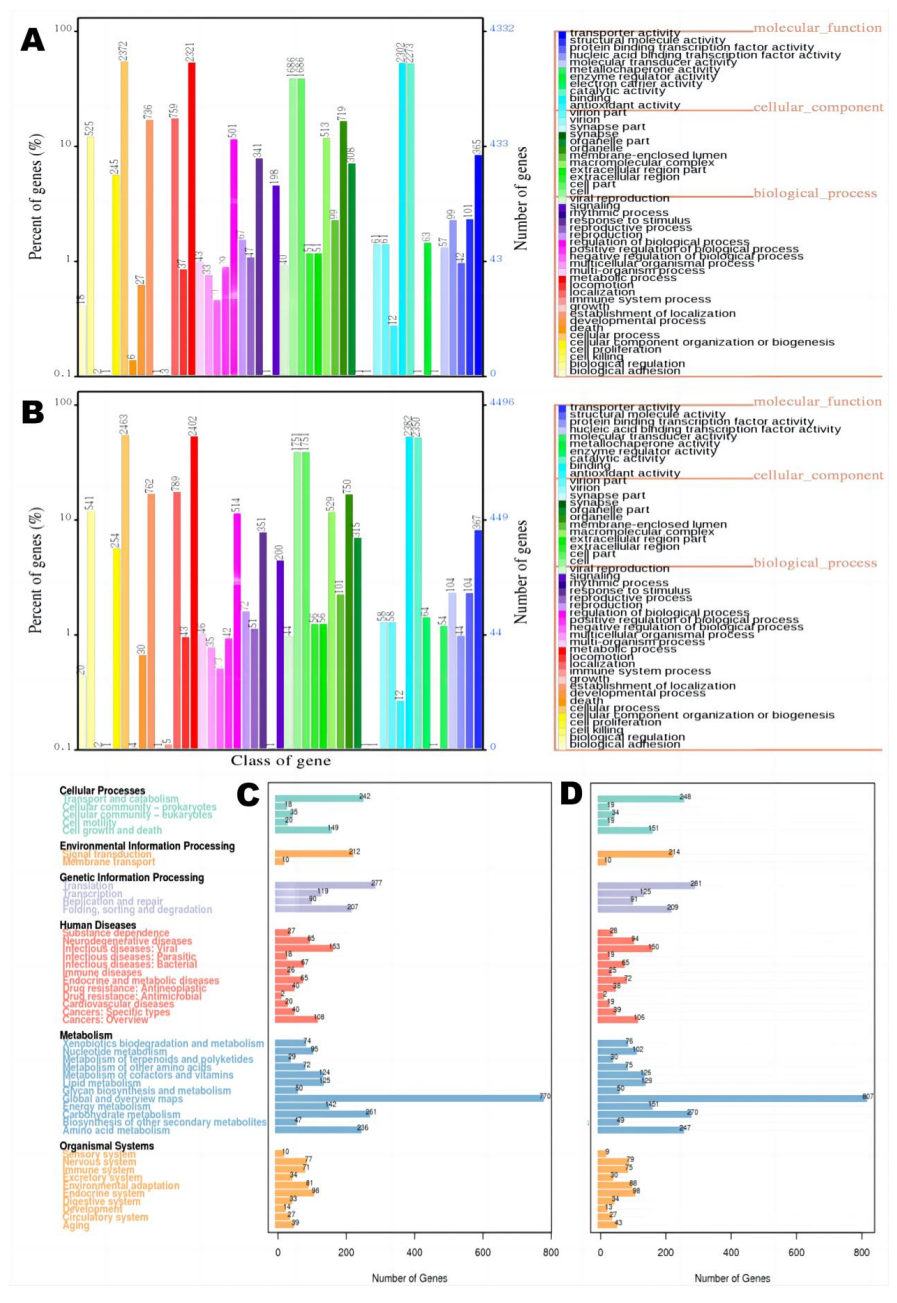
Functional classification of *M. purpureus* M3 and M34 gene annotations. Functional classification diagram of GO in (**A**) M3 and (**B**) M34 gene annotations; classification map of KEGG in (**C**) M3 and (**D**) M34 gene annotations.

Second, the main genome features (coding genes, repeat sequences, and non-coding RNA) of these fungi were predicted. In GO functional annotation, there were 5316 genes annotated with a molecular function and 11 functional gene classifications in M3, whereas 5482 genes annotated with a molecular function and 10 functional gene classifications were detected in M34. Gene A4420 was only detected in M3, which is predicted to play a role in metal ion transmembrane transport. There were 8383 (M3) and 8696 (M34) genes with biological processes annotation in 25 functional gene classifications. Among them, 2321 (M3) and 2402 (M34) genes may participate in metabolic processes, forming the most prominent group. A total of 5237 (M3) and 5427 (M34) genes were annotated as cell components in 12 functional gene classifications. All these numbers in M3 were similar to the number of genes identified in M34 (Figure 3A). In KEGG annotation, the largest number of genes was predicted to be involved in metabolic processes (2024 in M3 and 2112 in M34, Figure 3B). Based on the nonredundant Protein Database, the annotation results showed that *M. purpureus* was the most similar species with 5376 (M3) and 5649 (M34) matched genes (Figure S1). The KOG classification analysis for these two fungi showed that the number of genes within the twenty categories were similar to each other, with the largest number of genes found in the categories J (translation of ribosome structure and biogenesis), O (post-translational protein modification, turnover, and chaperones), and R (general function prediction only). In particularly, there were 41 (M3) and 38 (M34) genes in category Q (secondary metabolites biosynthesis, transport and catabolism) including type I PKSs (Figure S2). Meanwhile, the prevalent gene function annotations based on the TCDB Database were electrochemical potential-driven transporters (158 in M3 and 164 in M34) and primary active transporters (146 in M3 and 149 in M34) (Figure S3). The most frequent carbohydrate gene function annotations based on the CAZy Database were glycoside hydrolase (166 in M3 and 162 in M34) and glycosyltransferase (90 in M3 and 95 in M34, Figure S4).

### 3.4. Comparison of Secondary Metabolite Biosynthetic Gene Clusters in the Two Strains

**Figure 4:**
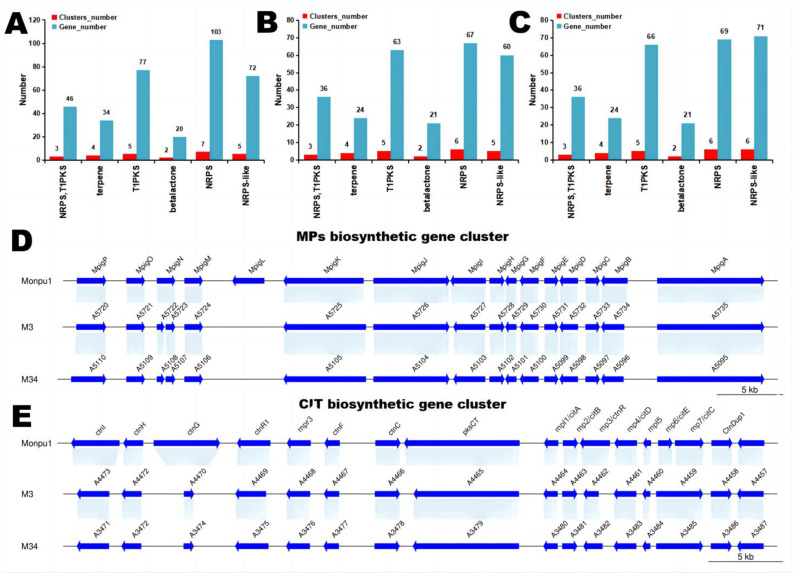
Numbers of BGCs belonging to major secondary metabolite categories and the numbers of the corresponding biosynthetic genes in (**A**) the reference genome Monpu1, (**B**) M3, and (**C**) M34. NRPS, T1PKS: hybrid nonribosomal peptide-polyketide BGC; T1PKS: type I iterative PKS-based BGC. BGCs of (**D**) MPs and (**E**) CIT from Monpu1, M3, and M34, respectively.

An analysis of the secondary metabolite biosynthetic capacities of the two strains revealed a similar number of BGCs that show a similar distribution among the main categories, which were also similar to the reference genome Monpu1 (Figure 4A–C). Thus, the secondary metabolisms of these two *M. purpureus* strains are dominated by nonribosomal peptides, polyketides, and their hybrids, as shown by the numbers of nonribosomal peptide synthetase (NRPS)-containing and PKS-containing BGCs. In particular, both strains M3 and M34 display five BGCs featuring type I iterative PKSs, the same as the reference genome Monpu1. The biosynthesis of MPs and CIT both require such PKSs. The total number of predicted biosynthetic genes in these PKS clusters were also found to be similar (63 and 66, respectively). The genes related to CIT and MPs synthesis in strains M3, M34, and Monpu1 are listed in Table S3. The BGCs of MPs and CIT of high MPs-producing M3 and M34 were similar to that in the reference genome Monpu1 (Figure 4D,E). Although gene *MpigL* was not detected in either M3 or M34, this would not affect the abilities of these two fungi to produce MPs in high yields. Both of these fungi had a *ctnG* gene (encoding carbonic anhydrase), each with a shorter length than that of Monpu1, but their BGCs of CIT were very similar. The results of the present study reveal that the high MPs-producing strains M3 and M34 have very similar BGCs. To explore the potential key genes responsible for the different CIT productivity of these two *M. purpureus* strains, we compared the gene expression between M34 (the experiment group) and M3 (the control group).

### 3.5. Transcriptome Sequencing Statistics, Quality Assessment, and Comparative Analysis

After quality control and data filtering, a total of 42.27 Gb of clean data was collected from the two strains in triplicates (six samples; generating 47.26–51.36 million raw reads representing 7.09–7.70 Gb clean data/sample; Table S4). The ratio of Q30 bases and valid bases were all higher than 94%, indicating a sufficient sequencing quality for further analysis. Moreover, the contents of the GC and AT of each sequencing read should be stable and equal at each position during the sequencing: the GC contents in all our samples were distributed around 52%, indicating highly accurate sequencing data. The clean reads of six samples were successfully mapped onto the reference genome (Monpu1) with the mapping ratios ranging from 92.22–97.76%.

**Figure 5:**
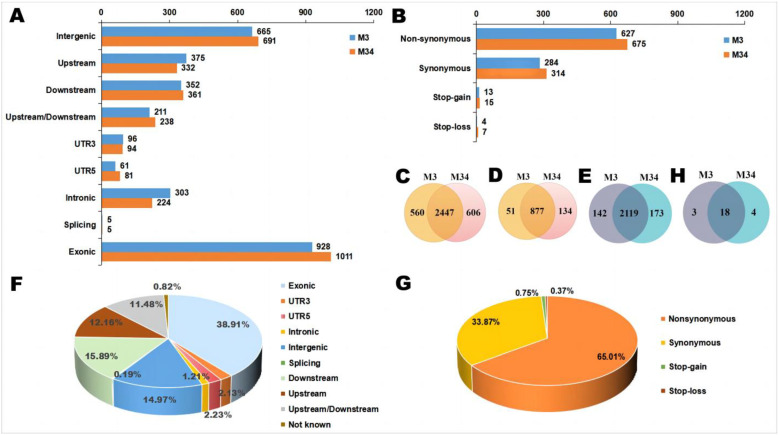
Location statistics of the SNPs in strains M3 and M34. (**A**) Distribution of genomes-based SNPs in different gene regions. UTR, untranslated region; SNP, single nucleotide polymorphism. (**B**) Variation information of the genomes-based SNPs located in the exonic regions. (**C**) Venn diagram of genomes-based SNPs. (**D**) Venn diagram of genomes-based SNPs located in the exonic regions. (**E**) Venn diagram of transcriptome-based SNPs. (**F**) Distribution of transcriptome-based SNPs in different gene regions. (**G**) Variation information of the transcriptome-based SNPs located in the exonic regions. (**H**) Venn diagram of transcriptome-based SNPs in primary metabolic genes.

SNPs are the most frequent type of variation in a genome. Upon mapping both the assembled genomes and the transcriptomic clean reads to the reference genome Monpu1 through BWA and SAMtools, a total of 3007 and 3053 SNPs were found in the M3 and M34 genomes, respectively. Overall, 2447 SNPs were common in these two fungi, while 560 or 606 SNPs were detected only in M3 or M34, respectively (Figure 5A–C, Tables S5 and S6). Most of the SNPs were in the exons: stop-gain, stop-loss, synonymous, and non-synonymous SNPs were detected in similar numbers in M3 and M34. In the exonic regions, 877 SNPs were common, while 51 or 134 SNPs were detected only in M3 or M34, respectively (Figure 5D). The rate of transition to transversion was 2.182 and 2.293, respectively.

Meanwhile, 2261 and 2292 SNPs were detected using the transcriptomic reads of M3 or M34, respectively. Among these, 2119 SNPs were common in the two strains, corresponding to a SNPs similarity of 89.79% (number of the common SNPs/number of the total SNPs; Figure 5E and Table S7). Among these 2119 shared SNPs, the number of transition substitutions was higher than that of transversions (79.91% vs. 20.09%, respectively). The genomic locations and the proportions of SNPs in the exon, UTR3, UTR5, intron, intergenic and splicing regions are presented in Figure 5F. Among the SNPs in the exon regions with both genomes and transcriptomic reads, more than 65% were nonsynonymous, while less than 1% represented stop-gains (Figure 5G).

Next, we compared the exon sequences of the genes in the CIT and MPs BGCs of strains M3 and M34 with those of the reference genome (Table 2). We found three SNPs in the MPs BGCs: all three were shared by strains M3 and M34 in both the assembled genomes and the transcriptomic clean reads. These shared SNPs were all nonsynonymous and located in *MpigA* (encoding the nrPKS), *MpigB* (transcription factor), and *MpigC* (C11-ketoreductase). The SNPs located in *MpigB* and *MpigC* were transitions (T/C), while the SNP located in *MpigA* was a transversion (A/C). Since both M3 and M34 are high producers of MPs, these shared SNPs apparently did not reduce their ability to produce MPs. In contrast, two nonsynonymous SNPs were detected in the CIT BGC of M34 only. These were located in *ctnR1* (encoding a WD repeat protein) and *citB* (oxygenase), and both of them were transitions (G/A). This suggests that these genes encode mutant versions of the WD repeat protein and the oxygenase enzyme, and may contribute to the low yields of CIT in M34. Furthermore, a total of 159 primary metabolic gene sequences from the two transcriptomes were randomly selected for comparison with the reference genome: 21 or 22 SNPs were detected in M3 or M34, respectively (Figure 5H). The SNPs similarity of these primary metabolic genes between strains M3 and M34 was 78.26% with 18 common SNPs. Therefore, the similarity of both the genome-based and the transcriptome-based SNPs between M3 and M34 was higher than 97%.

### 3.6. Functional Annotation of DEGs

**Figure 6:**
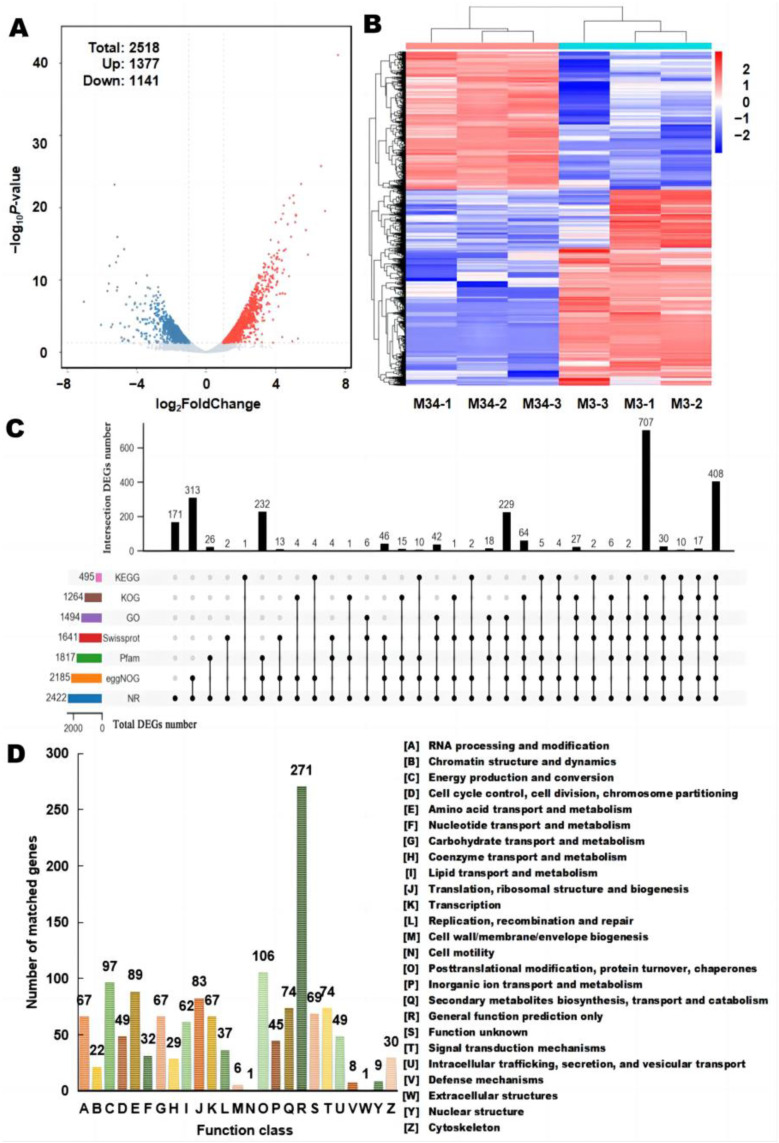
Comparative transcriptome analysis between *M. purpureus* M3 (the control group) and M34 (the experiment group). (**A**) Volcano plot analysis of DEGs; the red, green, and grey dots represent downregulated, upregulated, and not differentially expressed genes, respectively; the log_2_ FC on the *X*-axis represents the gene expression level, and the log_2_ *p* on the *Y*-axis shows the multiple of the expression difference. (**B**) Clustering of the DEGs. (**C**) UpSet plot of DEGs annotated using the seven databases. The horizontal bars represent the total number of DEGs in the listed databases. The vertical bars represent the number of DEGs commonly annotated among the databases that are indicated by black circles along the *X*-axis. A line linking multiple filled circles represents a column in which those DEGs are shared among the different functional databases. (**D**) KOG functional classification of DEGs.

Next, we conducted a comparative transcriptome analysis with the representative high MPs-producing, high CIT-producing *M. purpureus* M3 as the control and strain M34 (low CIT yield) as the experiment group. The analysis indicated that 2518 genes were differentially expressed, among which 1141 genes were downregulated and 1377 genes were upregulated in M34 (Figure 6A,B and Table S8). These genes are of particular interest for understanding the differences in transcriptional regulation between the two representative high MPs-producing strains. These 2518 DEGs were functionally annotated using seven databases, including the GenBank non-redundant protein (96.19%), Swiss-Prot (65.17%), KOG (50.20%), eggNOG (86.78%), GO (59.37%), KEGG (19.66%) and Pfam (72.16%) databases. Intersections of these annotations are graphically represented in Figure 6C as an UpSet plot. In particular, 408 of the DEGs were commonly annotated in all databases.

#### 3.6.1. KOG Functional Annotation

Using the KOG database, 1264 of the 2518 DEGs were assigned to 25 functional categories, while the remaining 1254 DEGs matched no KOG annotations (Figure 6D). Most DEGs belong to the functional categories R (General function prediction only, 21.44%), O (Post-translational modification, protein turnover, chaperones, 8.39%), C (Energy production and conversion, 7.67%), E (Amino acid transport and metabolism, 7.07%), and J (Translation, ribosomal structure, and biogenesis, 6.57%). Importantly, 74 DEGs were annotated as class Q (Secondary metabolites biosynthesis, transport, and catabolism, 5.85%), which need to be further analyzed with other functional databases.

#### 3.6.2. GO Functional Annotation Analysis

**Figure 7:**
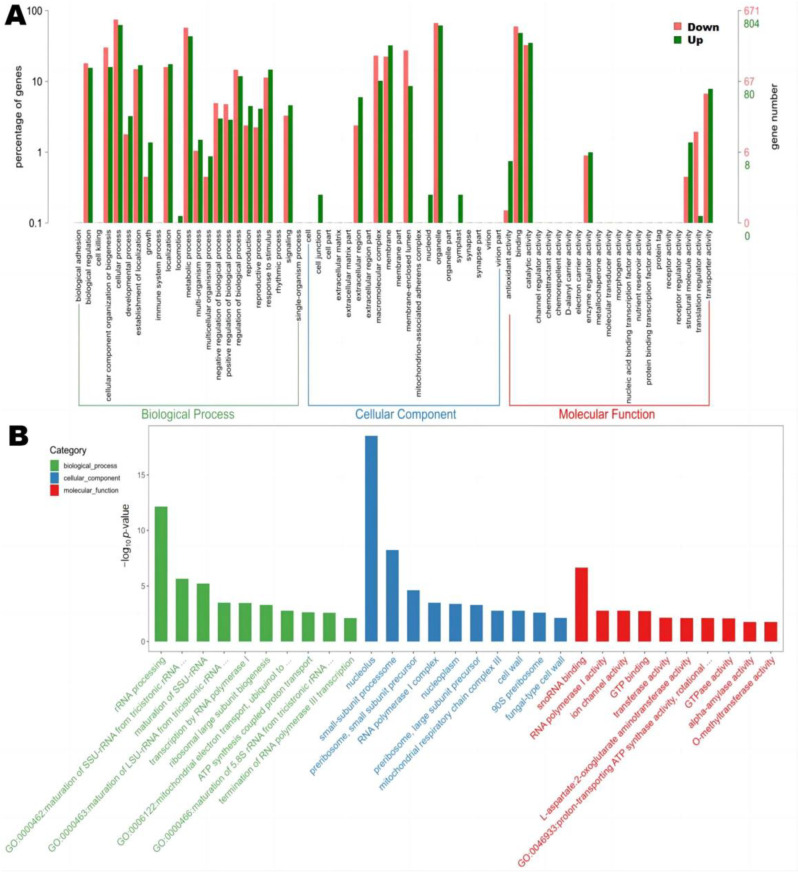
Functional classification of DEGs in strains M3 and M34. (**A**) GO functional classification of DEGs. Red bars represent the number of downregulated DEGs; green bars show upregulated DEGs. (**B**) The top 30 GO enrichment terms from M34 vs. M3. The green, blue and red bars represent the biological process, cellular component, and molecular function, respectively.

A GO enrichment analysis of all DEGs was performed to elucidate the biological functions of DEGs with significant changes in expression levels (*p*  < 0.05). A total of 1475 DEGs were classified into three GO categories and 33 functional subcategories, including biological processes (BP: 18 groups), cellular components (CC: 8 groups), and molecular functions (MF: 7 groups) (Figure 7A). The top 30 terms of GO enrichment analyses show that the terms rRNA processing (GO:0006364), nucleolus (GO:0005730), and snoRNA binding (GO:0030515) were the most represented for the BP, CC, and MF category, respectively (Figure 7B). They were all significantly downregulated in M34, indicating reduced protein coding and synthesis relative to M3 (Figure S5A). The secondary metabolites of M34 are synthesized at lower amounts than those in M3, requiring less protein synthesis during secondary metabolism. Additionally, the upregulated DEGs were mainly involved in ATP synthesis-coupled proton transport (GO:0015986) and the oxidation-reduction process (GO:0055114) in the BP ontology; fungal-type cell wall (GO:0009277) in the CC ontology; and L-aspartate: 2-oxoglutarate aminotransferase activity (GO:0004069) in the MF ontology (Figure S5B). The transcription levels of 12 genes involved in oxidation-reduction processes were significantly increased, which might result in an increase in energy accumulation upon reduced biosynthesis of CIT [14]. The results also suggested that the organelles and enzymes involved in protein secretion influence the secondary metabolite synthesis pathways.

#### 3.6.3. KEGG Pathway Enrichment

**Figure 8:**
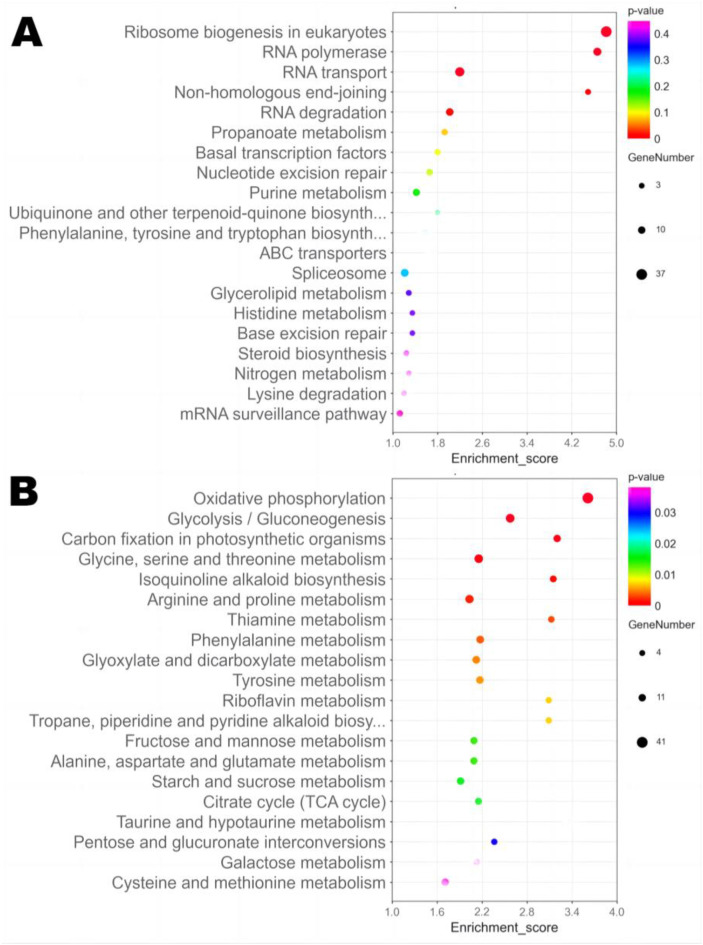
KEGG enrichment analysis of DEGs in *M. purpureus* strains M34 vs. M3. Scatter plot of the top 20 KEGG pathways enriched by downregulated DEGs (**A**) or upregulated DEGs (**B**) in strain M34. The size of the dot indicates the number of DEGs, and a smaller *p*-value indicates a more significant enrichment.

An analysis of the KEGG metabolic pathway enrichment classified 616 DEGs into six main categories with 19 subcategories, with significant enrichment in two categories: metabolism (carbohydrate metabolism, amino acid metabolism, and energy metabolism) and genetic information processing (translation) (*p* < 0.05, Figure S6A,B). Interestingly, the downregulated DEGs in M34 were significantly enriched in the category of genetic information processing, including ribosome biogenesis in eukaryotes (ko03008) and RNA polymerase (ko03020) (Figure 8A). The upregulated DEGs in strain M34 were all significantly enriched in the category of primary metabolism-related pathways, including oxidative phosphorylation (ko00190), glycolysis/gluconeogenesis (ko00010), and carbon fixation in photosynthetic organisms (ko00710), etc. (Figure 8B). In addition, the upregulated DEGs in M34 were significantly enriched in many amino acid metabolism-related pathways, including glycine, serine and threonine metabolism (ko00260), arginine and proline metabolism (ko00330), phenylalanine metabolism (ko00360), tyrosine metabolism (ko00350), alanine, and aspartate and glutamate metabolism (ko00250). These results indicate that the primary metabolic processes for energy, carbohydrate and amino acids metabolism are highly associated with CIT and MPs biosynthesis.

### 3.7. Analysis of the DEGs Involved in the Secondary Metabolisms of Strains M3 and M34

#### 3.7.1. Differential Gene Expression in the CIT BGC

**Figure 9:**
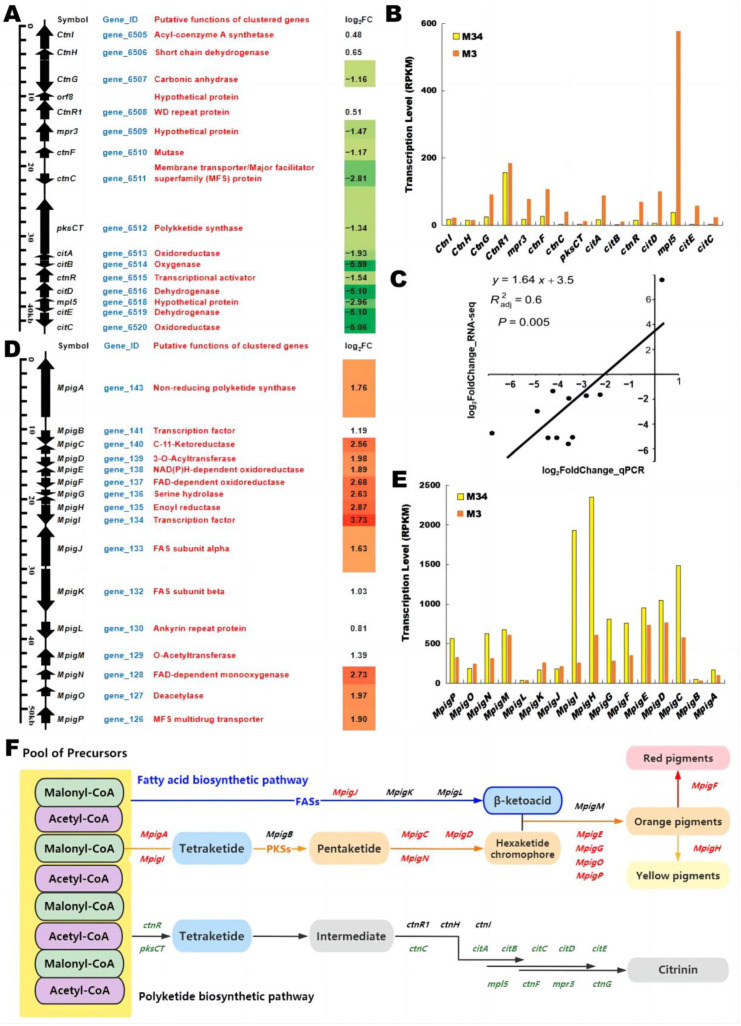
Differential expression of the MPs and CIT BGCs in strain M34 vs. M3. (**A**) The CIT BGCs with proposed functions of the encoded proteins in *M. purpureus* and expression log_2_FC of DEGs. (**B**) Transcription levels of DEGs involved in CIT biosynthesis. (**C**) Relative expression levels of CIT biosynthetic genes as monitored by qRT-PCR. (**D**) The MPs BGCs with proposed functions of the encoded proteins in *M. purpureus* and expression log_2_FC of DEGs. (**E**) Transcription levels of DEGs involved in MPs biosynthesis. (**F**) Integration map of the MPs and CIT biosynthetic pathways with relative DEGs in M34 vs. M3. Green represents the downregulated genes, red shows the upregulated genes, and black indicates genes with no significant changes.

As expected, the majority of the CIT biosynthesis genes were differentially expressed in *M. purpureus* M34 vs. M3. Compared to the high CIT-producing *M. purpureus* M3 (the control group), the results showed that 12 genes of the CIT BGC were significantly downregulated in the low CIT-producing *M. purpureus* M34 (the experiment group; Figure 9A,B, Table S9) [33]. These results indicate that the downregulation of the transcription level of these genes is contributing to the decrease of CIT biosynthesis. To further validate this result, the expression levels of the core CIT biosynthetic genes (*citC-ctnF*, the homologs of *mpl7-mpr2* in *M. purpureus*) and the global regulator *laeA* (gene_7831, 7.57 log_2_FC) were also analyzed by RT-qPCR (Figure 9C). The results showed that all the selected genes were significantly downregulated in M34 (*p* < 0.05), confirming the reliability of the RNA-Seq data. It is interesting to note that the genes *citB*, *citD, citE,* and *citC* exhibited the lowest log_2_FC (fold change) values (−5.59, −5.10, −5.10, and −5.06, respectively) in M34 relative to that in M3 (Figure 9A). This suggests that, consistent with previous results [34], the reduced expression of the oxidoreductase enzymes CitB, CitD and CitE, and the membrane transporter CitC, might be the most important contributors to the low CIT yield in M34. The most highly transcribed gene in the CIT BGC of M3, *mpl5* encoding a hypothetical protein, displays a log_2_FC of −2.96. In particular, the regulatory gene *ctnR* encoding a transcriptional activator and the *pksCT* encoding the non-reducing polyketide synthase (nrPKS) exhibited relatively low expression levels even in strain M3, and their transcription levels were further reduced in strain M34 by 0.34- and 0.39-fold, respectively (Table S9). The gene *pksCT* encodes the nrPKS that produces the CIT polyketide backbone. Previous studies reported that MPs production may be increased and CIT yield may be depressed in *M. purpureus* fermentation by applying low-frequency magnetic fields, and showed that the relative expression levels of *pksCT* and *ctnA* significantly decreased [35]. *He and Cox* [36] found that the heterologous co-expression of *citS* (the homolog of *pksCT*) and the *citA-E* genes in *A. oryzae* affords high amounts of CIT. Conversely, the deletion of *citE*, *citS*, *citB*, and *citA*, respectively, results in a dramatically reduced CIT yield, but has little effect on MPs production [37,38]. Previous reports indicated that the genes *ctnF* and *ctnG* were associated with the accumulation of acetyl-CoA and malonyl-CoA (the common precursors for MPs and CIT biosynthesis), and their expression improves both CIT and MPs biosynthesis [39]. Both of these genes were downregulated in strain M34. Our SNP analysis (Section 3.4) revealed non-synonymous replacements in *ctnR1* (encoding a WD repeat protein) and *citB* (encoding an oxygenase) in strain M34. However, *ctnR1* (just as two other CIT BGC genes, *ctnI* (acyl-CoA synthetase) and *ctnH* (short chain dehydrogenase)) were not differentially expressed in the two strains (*p* > 0.05). In contrast, the gene *citB*, which features a non-synonymous SNP and showed one of the highest reductions in its transcription level in strain M34, might be strongly associated with the low CIT productivity of that strain. Thus, the role of *citB* in the reduction of CIT production in strain M34 needs to be investigated further.

#### 3.7.2. Differential Gene Expression Related to MPs

MPs are a mixture of yellow, orange, and red azaphilone pigments. The MPs BGC is a composite supercluster that is responsible not only for the production of azaphilones, but also the biosynthesis of the monasone-type naphthoquinones [40]. The MPs BGC includes 16 highly conserved genes from *MpigA*-*MpigP* (gene_126-143, Figure 9D,E) [41,42]. Four genes in the MPs BGC (*MpigK* (fatty acid synthesis-related gene), *MpigL* (ankyrin repeat protein), *MpigM* (*O*-acetyltransferase), and *MpigB* (positive regulator)) were not differentially expressed in M34 relative to those in M3 (*p* > 0.05). However, the expression levels of 12 genes in this BGC (*MpigP*, *MpigO*, *MpigN*, *MpigJ*, *MpigI*, *MpigH*, *MpigG*, *MpigF*, *MpigE*, *MpigD*, *MpigC*, and *MpigA*) were higher in strain M34 compared to those in strain M3 (*p* < 0.05), with log_2_FC values ranging from 3.73 to 1.76 (Figure 9D, Table S9). Considering that M34 produced somewhat less MPs than strain M3 (Figure 1), this observation is surprising. We hypothesize that strain M34 needs to compensate with its higher level of expression of these genes in the MPs BGC for certain, yet-unknown metabolic deficiencies in order to be able to approximate the MPs productivity of strain M3. Proteomic and metabolomic analyses of strains M34 and M3 may shed more light on this discrepancy in the future. An alternative hypothesis may be that the transcription factor *MpigI* (gene_134) that is overexpressed in strain M34 (log_2_FC > 3) not only leads to the overexpression of the MPs BGC, but may also negatively regulate the expression of the CIT BGC (Figure 9F). Such cross-talk at the level of transcription regulators is often encountered in filamentous fungi [43].

### 3.8. Differential Gene Expression of Transcription Factors

Transcription factors (TFs) can regulate polyketide production by modulating the expression of genes in the BGCs, or they may affect the overall metabolism of the cell by controlling different biological processes [44]. Based on Pfam annotations and similarities with entries in the GenBank nonredundant protein database, there are 233 TF-encoding genes in the *M. purpureus* genome. Among these, 93 were DEGs, with 50 TFs downregulated and 43 TFs upregulated in strain M34 compared to strain M3 (Table S10). This indicates a potentially wide-ranging restructuring of the metabolism of strain M34, as is already evident from the large number of genes (2518) that were found to be differentially transcribed in M34 vs. M3 (Section 3.5). In particular, 42 of these differentially expressed TFs were annotated as “Fungal Zn(2)-Cys(6) binuclear cluster domain proteins”, with 24 and 18 DEGs exhibiting reduced or increased transcription levels in strain M34, respectively (Figure S7). The Zn(2)-Cys(6) binuclear cluster domain transcriptional regulators play essential roles in secondary metabolite biosynthesis. Compared to M3, the transcription levels of *ctnR* (gene_6515, encoding the citrinin biosynthesis Zn(2)-Cys(6) binuclear cluster domain transcriptional activator from the CIT BGC) was downregulated (1.54 log_2_FC), whereas *MpigI* (gene_134, encoding a transcriptional regulator from the MPs BGC) was upregulated (3.73 log_2_FC) in M34, as noted earlier (Section 3.6.2). Therefore, the high expression of *MpigI* and low expression of *ctnR* may lead *M. purpureus* to afford high MPs and low CIT yields. These differentially expressed TFs represent prime candidates to further decipher the multilevel regulation and complex interplay of CIT and MPs biosynthesis in *M. purpureus*.

## 4. Conclusions

MPs have been widely used as food colorants and preservatives, nutraceuticals, ingredients in cosmetics, and as pigments for wide-ranging industrial applications. However, due to the potential presence of citrinin, the safety of MPs-containing products needs to be carefully evaluated and monitored. Recent research to improve MPs production and inhibit CIT production has mainly focused on medium optimization and fermentation process development. The key factor for the industrial production of MPs without high levels of CIT contamination is to isolate *Monascus* strains that inherently produce little or no CIT. In this work, high MPs-producing strains of *M. purpureus* with extremely high (M3) vs. low CIT yield (M34) were identified. A comparative transcriptomic analysis was performed to explore the impact of the genetic differences in these strains on their global expression profiles, and to recognize potential key genes that participate in, regulate, or indirectly influence the production of CIT. This transcriptome analysis highlighted the differences in the expression of the genes located in the MPs and CIT BGCs in *M. purpureus*. Further, this analysis identified a large number of genes that were differentially expressed between the two strains, including putative transcriptional regulators that may be responsible for the different transcriptional profiles of these strains. This investigation uncovered potential key rate-limiting genes that may be modulated by genetic engineering to further reduce or even eliminate CIT production. Further studies are recommended to focus on integrating transcriptome and proteome analyses, and enlist untargeted metabolomics, during different stages of MPs fermentations. Such results would be especially important to further improve the safety of MPs-containing products used in the food industry.

## Figures and Tables

**Table 1:** General features of *M. purpureus* M3 and M34 genomes.

Sample	Total Scaffold Number (>500 bp)	Total Contig Number (>500 bp)	Sequence GC (%)	Genome Size (bp)	Gene Number	Total Gene Length (bp)	tRNA Number
M3	262	422	48.99	23,271,143	6062	9,013,874	128
M34	295	449	49.00	23,263,192	6312	9,334,696	138

**Table 2:** Genome-based and transcriptome-based SNPs in the CIT and MPs BGCs of *M. purpureus* M3 and M34.

Genes	Chromosome	Position	Ref	Alt	Qual Value	NR Annotation	Location	Type
*MpigC*	scaffold_1	351,367	T	C	518,198.55	C-11-ketoreductase	M3 and M34	Nonsynonymous
*MpigB*	scaffold_1	352,990	T	C	85,333.55	transcription factor	M3 and M34	Nonsynonymous
*MpigB*	scaffold_1	353,255	A	G	353,255.00	transcription factor	M3 and M34	Synonymous
*MpigB*	scaffold_1	353,748	C	T	353,748.00	transcription factor	M3 and M34	Synonymous
*MpigA*	scaffold_1	357,147	A	C	38,461.55	nrPKS	M3 and M34	Nonsynonymous
*ctnR1*	scaffold_83	60,975	G	A	79,212.49	CtnR1 WD repeat	M34	Nonsynonymous
*citB*	scaffold_83	83,512	G	A	5595.49	CitB oxygenase	M34	Nonsynonymous

## Data Availability

The raw data supporting the conclusions of this manuscript will be made available by the authors, without undue reservation, to any qualified researcher. The raw RNA-seq data of the present study was deposited into the NCBI database with an accession number of PRJNA862287.

## Supplementary Materials

The following supporting information can be downloaded at: https://www.mdpi.com/article/10.3390/jof9020200/s1, Figure S1: Species annotation statistics from the NR database of (A) M3 and (B) M34; Figure S2: Functional classification diagram of KOG in (A) M3 and (B) M34 gene annotations; Figure S3: Functional classification diagram of TCDB in (A) M3 and (B) M34 gene annotations; Figure S4: Functional classification diagram of CAZy in (A) M3 and (B) M34 gene annotations; Figure S5: Functional classification of DEGs in strains M3 and M34. Scatter plot of the top 20 GO terms enriched in the downregulated (A) and the upregulated (B) DEGs in M34; Figure S6: KEGG enrichment analysis of DEGs in *M. purpureus* strains M34 vs. M3. (A) KEGG enrichment pathways of DEGs. (B) The top 20 pathways in the KEGG enrichment analysis of DEGs; Figure S7: Expression fold changes (log_2_FC) of selected DEGs that encode fungal Zn(2)-Cys(6) binuclear cluster domain proteins; Table S1: qRT-PCR primers used in this study; Table S2: ITS taxonomic characterization of *Monascus* strains; Table S3: The genes in the CIT and MPs biosynthetic gene clusters from M3 and M34, compared to the reference genome Monpu1, respectively; Table S4: Quality evaluation of the transcriptome data; Table S5: Genome-based SNPs analysis in M3; Table S6: Genome-based SNPs analysis in M34; Table S7: Transcriptome-based SNPs analysis; Table S8: The profiles of RNA-seq reads mapped to the genome of *Monascus purpureus* and the differential expression analysis (strain M34 vs. strain M3); Table S9: The expression profiles of genes involved in CIT and MPs biosynthesis in strain M34 vs. strain M3; Table S10: Expression profiles of transcription factor DEGs with differential transcriptional levels in *M. purpureus* M34, compared to that in the M3 strain.

## References

[1] Huang Y.-Y., Liang Z.-C., Lin X.-Z., He Z.-G., Ren X.-Y., Li W.-X., Molnár I. (2021). Fungal community diversity and fermentation characteristics in regional varieties of traditional fermentation starters for Hong Qu glutinous rice wine. Food Res. Int..

[2] Feng S.-S., Li W., Hu Y.-J., Feng J.-X., Deng J. (2022). The biological activity and application of *Monascus* pigments: A mini review. Int. J. Food Eng..

[3] Chai X., Ai Z., Liu J., Guo T., Wu J., Bai J., Lin Q. (2020). Effects of pigment and citrinin biosynthesis on the metabolism and morphology of *Monascus purpureus* in submerged fermentation. Food Sci. Biotechnol..

[4] He J., Jia M., Li W., Deng J., Ren J., Luo F., Bai J., Liu J. (2021). Toward improvements for enhancement the productivity and color value of *Monascus* pigments: A critical *review* with recent updates. Crit. Rev. Food Sci. Nutr..

[5] Farawahida A.H., Palmer J., Flint S. (2022). *Monascus* spp. and citrinin: Identification, selection of *Monascus* spp. isolates, occurrence, detection and reduction of citrinin during the fermentation of red fermented rice. Int. J. Food Microbiol..

[6] Silva L.J.G., Pereira A.T., Pena A., Lino C.M. (2020). Citrinin in Foods and Supplements: A Review of Occurrence and Analytical Methodologies. Foods.

[7] Shi J., Qin X., Zhao Y., Sun X., Yu X., Feng Y. (2022). Strategies to enhance the production efficiency of Monascus pigments and control citrinin contamination. Process. Biochem..

[8] Wei S., He Y., Yang J., Li Y., Liu Z., Wang W. (2022). Effects of exogenous ascorbic acid on yields of citrinin and pigments, antioxidant capacities, and fatty acid composition of Monascus ruber. Lwt.

[9] Liu H.-Q., Huang Z.-F., Yang S.-Z., Tian X.-F., Wu Z.-Q. (2021). Inducing red pigment and inhibiting citrinin production by adding lanthanum(III) ion in *Monascus purpureus* fermentation. Appl. Microbiol. Biotechnol..

[10] Liu W., An C.-Y., Shu X., Meng X., Yao Y., Zhang J., Chen F., Xiang H., Yang S., Gao X. (2020). A Dual-Plasmid CRISPR/Cas System for Mycotoxin Elimination in Polykaryotic Industrial Fungi. ACS Synth. Biol..

[11] Ghosh S., Dam B. (2020). Genome shuffling improves pigment and other bioactive compound production in *Monascus purpureus*. Appl. Microbiol. Biotechnol..

[12] Li W.-L., Hong J.-L., Lu J.-Q., Tong S.-G., Ni L., Liu B., Lv X.-C. (2022). Comparative Transcriptomic and Metabolomic Analyses Reveal the Regulatory Effect and Mechanism of Tea Extracts on the Biosynthesis of *Monascus* Pigments. Foods.

[13] Huang D., Wang Y., Zhang J., Xu H., Bai J., Zhang H., Jiang X., Yuan J., Lu G., Jiang L. (2021). Integrative Metabolomic and Transcriptomic Analyses Uncover Metabolic Alterations and Pigment Diversity in *Monascus* in Response to Different Nitrogen Sources. Msystems.

[14] Shi J., Zhao W., Lu J., Wang W., Yu X., Feng Y. (2021). Insight into Monascus pigments production promoted by glycerol based on physiological and transcriptome analyses. Process. Biochem..

[15] Tong A., Lu J., Huang Z., Huang Q., Zhang Y., Farag M.A., Liu B., Zhao C. (2022). Comparative transcriptomics discloses the regulatory impact of carbon/nitrogen fermentation on the biosynthesis of *Monascus kaoliang* pigments. Food Chem. X.

[16] Ouyang W., Liu X., Wang Y., Huang Z., Li X. (2021). Addition of genistein to the fermentation process reduces citrinin production by *Monascus* via changes at the transcription level. Food Chem..

[17] Qiao J., He X., Wang C., Yang H., Xin Z., Xin B., Wang J., Dong R., Zeng H., Li F. (2022). Transcriptome analysis revealing molecular mechanisms of enhanced pigment yield by succinic acid and fluconazole. Prep. Biochem. Biotechnol..

[18] Hong J.-L., Wu L., Lu J.-Q., Zhou W.-B., Cao Y.-J., Lv W.-L., Liu B., Rao P.-F., Ni L., Lv X.-C. (2020). Comparative transcriptomic analysis reveals the regulatory effects of inorganic nitrogen on the biosynthesis of *Monascus* pigments and citrinin. RSC Adv..

[19] Liang B., Du X.-J., Li P., Sun C.-C., Wang S. (2018). Investigation of Citrinin and Pigment Biosynthesis Mechanisms in *Monascus purpureus* by Transcriptomic Analysis. Front. Microbiol..

[20] Yang C., Wu X., Chen B., Deng S., Chen Z., Huang Y., Jin S. (2017). Comparative analysis of genetic polymorphisms among *Monascus* strains by ISSR and RAPD markers. J. Sci. Food Agric..

[21] Chen S., Huang Y., Lu D., Yang C. (2018). Effects of carbon and nitrogen sources on production of L, Idu pigments and citrinin in different characteristics of Monascus strains. China Condiment.

[22] Yang C., Chen Z., Wu X., Deng S., Huang Y., Lu D. (2015). Genomic analysis of ITS sequence for classification and determination of Monascus strains. J. Nucl. Agric. Sci..

[23] Lim H.J., Lee E., Yoon Y., Chua B., Son A. (2016). Portable lysis apparatus for rapid single-step DNA extraction of *Bacillus subtilis*. J. Appl. Microbiol..

[24] Li R., Zhu H., Ruan J., Qian W., Fang X., Shi Z., Li Y., Li S., Shan G., Kristiansen K. (2010). De novo assembly of human genomes with massively parallel short read sequencing. Genome Res..

[25] Li R., Li Y., Kristiansen K., Wang J. (2008). SOAP: Short oligonucleotide alignment program. Bioinformatics.

[26] Bankevich A., Nurk S., Antipov D., Gurevich A.A., Dvorkin M., Kulikov A.S., Lesin V.M., Nikolenko S.I., Pham S., Prjibelski A.D. (2012). SPAdes: A new genome assembly algorithm and its applications to single-cell sequencing. J. Comput. Biol..

[27] Simpson J.T., Wong K., Jackman S.D., Schein J.E., Jones S.J., Birol I. (2009). ABySS: A parallel assembler for short read sequence data. Genome Res..

[28] Xie L., Xie J., Chen X., Tao X., Xie J., Shi X., Huang Z. (2022). Comparative transcriptome analysis of *Monascus purpureus* at different fermentation times revealed candidate genes involved in exopolysaccharide biosynthesis. Food Res. Int..

[29] Li H., Durbin R. (2009). Fast and accurate short read alignment with Burrows—Wheeler transform. Bioinformatics.

[30] McKenna A., Hanna M., Banks E., Sivachenko A., Cibulskis K., Kernytsky A., Garimella K., Altshuler D., Gabriel S., Daly M. (2010). The Genome Analysis Toolkit: A MapReduce framework for analyzing next-generation DNA sequencing data. Genome Res..

[31] Wang K., Li M., Hakonarson H. (2010). ANNOVAR: Functional annotation of genetic variants from high-throughput sequencing data. Nucleic Acids Res..

[32] Medema M.H., Blin K., Cimermancic P., De Jager V., Zakrzewski P., Fischbach M.A., Weber T., Takano E., Breitling R. (2011). antiSMASH: Rapid identification, annotation and analysis of secondary metabolite biosynthesis gene clusters in bacterial and fungal genome sequences. Nucleic Acids Res..

[33] Shimizu T., Kinoshita H., Nihira T. (2007). Identification and in vivo functional analysis by gene disruption of ctnA, an activator gene involved in citrinin biosynthesis in Monascus purpureus. Appl. Environ. Microb..

[34] Liang B., Du X., Li P., Guo H., Sun C., Gao J., Wang S. (2017). Orf6 gene encoded glyoxalase involved in mycotoxin citrinin biosynthesis in Monascus purpureus YY-1. Appl. Microbiol. Biotechnol..

[35] Wan Y., Zhang J., Han H., Li L., Liu Y., Gao M. (2017). Citrinin-producing capacity of *Monascus purpureus* in response to low−frequency magnetic fields. Process. Biochem..

[36] He Y., Cox R.J. (2016). The molecular steps of citrinin biosynthesis in fungi. Chem. Sci..

[37] Li Y., Wang N., Jiao X., Tu Z., He Q., Fu J. (2020). The ctnF gene is involved in citrinin and pigment synthesis in *Monascus aurantiacus*. J. Basic Microb..

[38] Ning Z.-Q., Cui H., Xu Y., Huang Z.-B., Tu Z., Li Y.-P. (2017). Deleting the citrinin biosynthesis-related gene, ctnE, to greatly reduce citrinin production in *Monascus aurantiacus* Li AS3.4384. Int. J. Food Microbiol..

[39] Li Y., Tang X., Wu W., Xu Y., Huang Z., He Q. (2015). The ctnG gene encodes carbonic anhydrase involved in mycotoxin citrinin biosynthesis from *Monascus aurantiacus*. Food Addit. Contam. Part A.

[40] Li M., Kang L., Ding X., Liu J., Liu Q., Shao Y., Molnár I., Chen F. (2020). Monasone naphthoquinone biosynthesis and resistance in Monascus fungi. mBio.

[41] Li L., Chen F. (2020). Effects of *mrpigG* on Development and Secondary Metabolism of *Monascus ruber* M7. J. Fungi.

[42] Chen W., Feng Y., Molnár I., Chen F. (2019). Nature and nurture: Confluence of pathway determinism with metabolic and chemical serendipity diversifies *Monascus* azaphilone pigments. Nat. Prod. Rep..

[43] Hidalgo P.I., Poirier E., Ullán R.V., Piqueras J., Meslet-Cladière L., Coton E., Coton M. (2017). Penicillium roqueforti PR toxin gene cluster characterization. Appl. Microbiol. Biotechnol..

[44] Liu J., Wu J., Cai X., Zhang S., Liang Y., Lin Q. (2020). Regulation of secondary metabolite biosynthesis in *Monascus purpureus* via cofactor metabolic engineering strategies. Food Microbiol..

