# Supplementary material for: Comparative Transcriptomic Analysis of Key Genes Involved in Citrinin Biosynthesis in Monascus purpureus
Source: J Fungi (Basel). 2023 Feb 3;9(2):200. doi: 10.3390/jof9020200 (PMC9965497; doi:10.3390/jof9020200)
Supplement: Supplementary file 1 [file jof-09-00200-s001.zip › jof-2150824-supplementary/SuppIementary Figures.pdf]

SI Figures

A

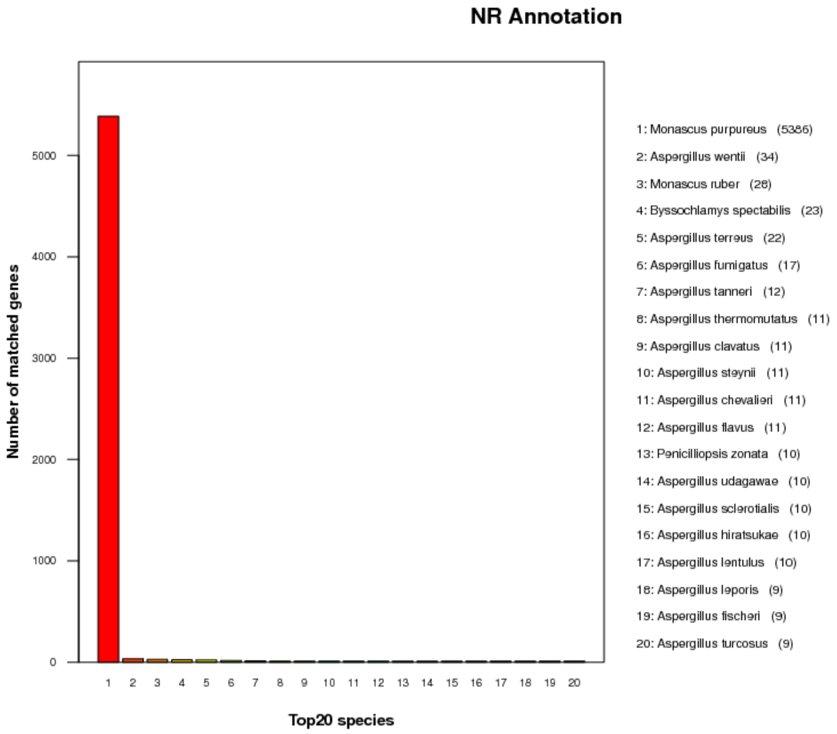

B

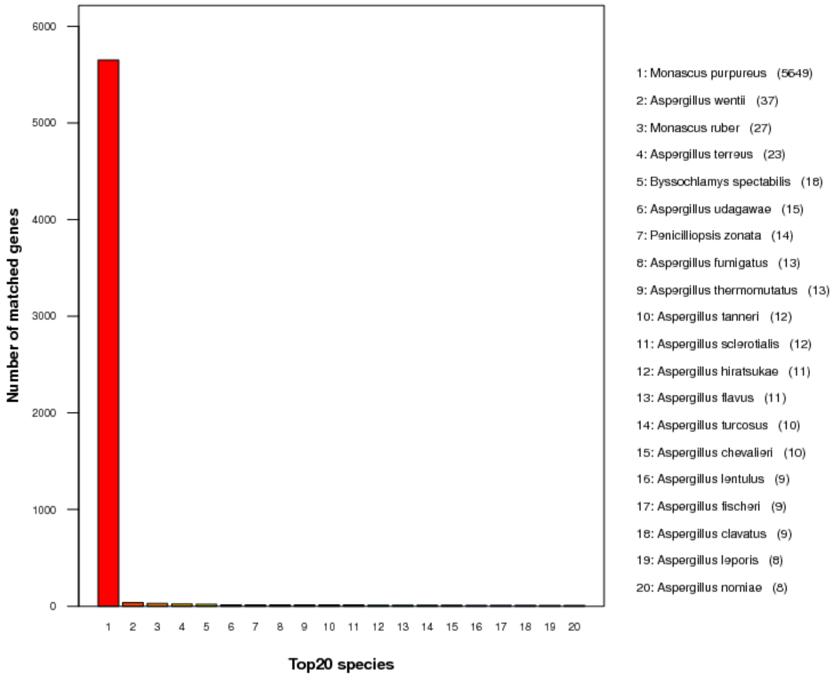

Figure S1. Species annotation statistics from the NR database of (A) M3 and (B) M34.

A

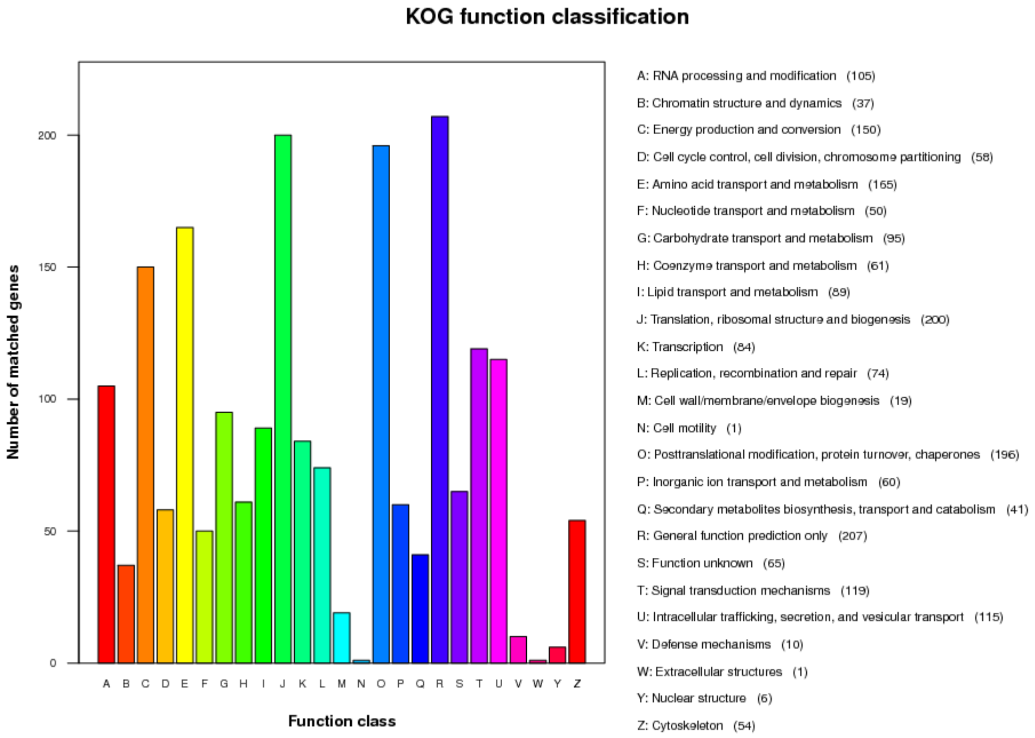

B

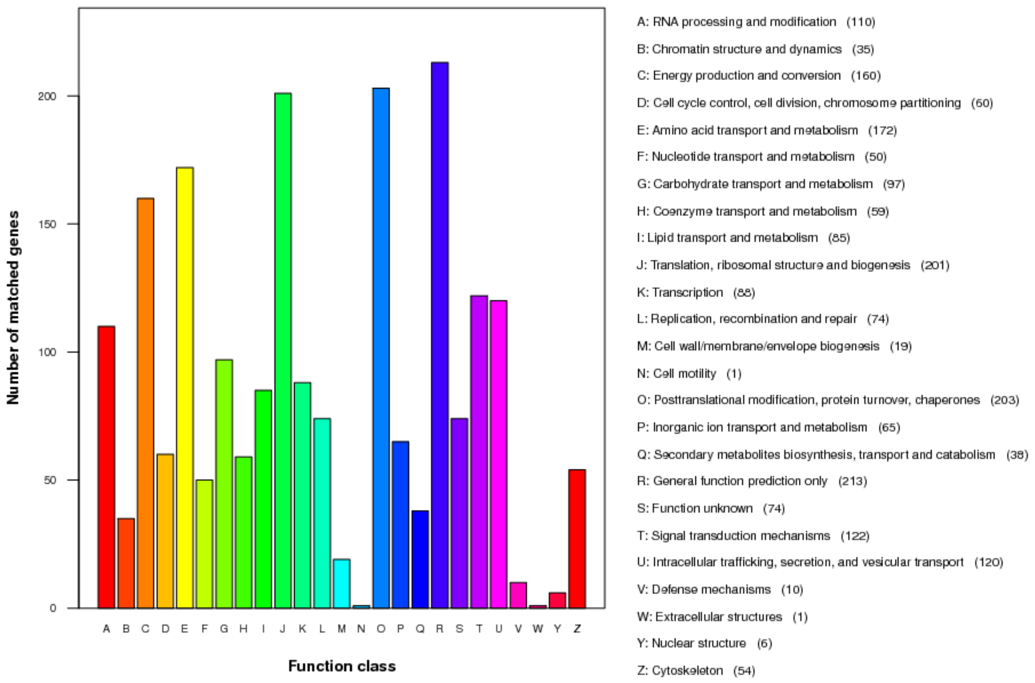

Figure S2. Functional classification diagram of KOG in (A) M3 and (B) M34 gene annotations.

A

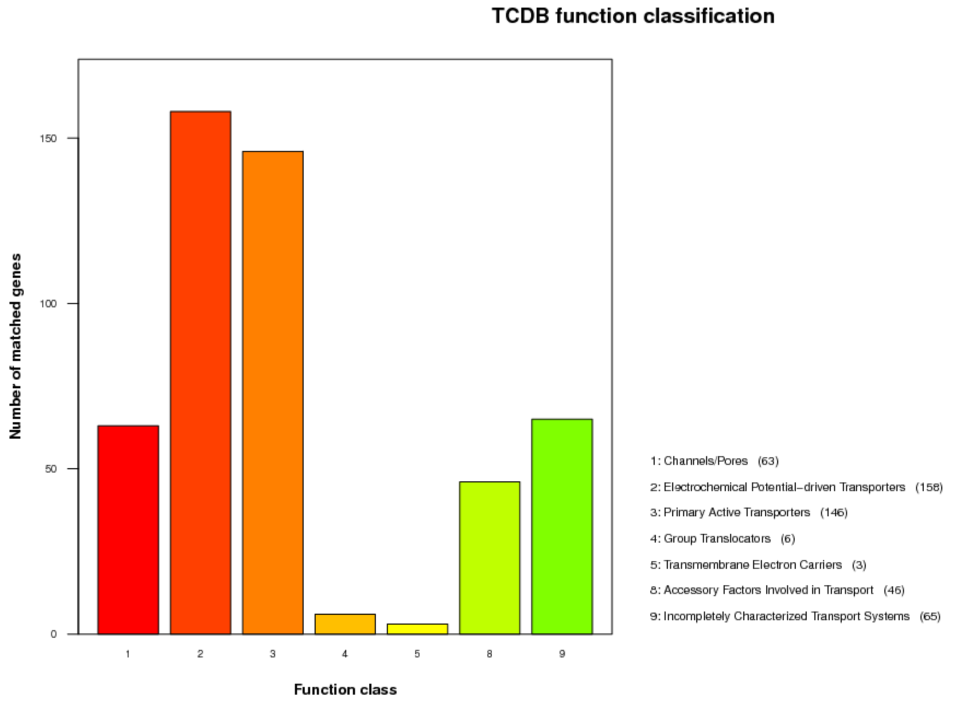

B

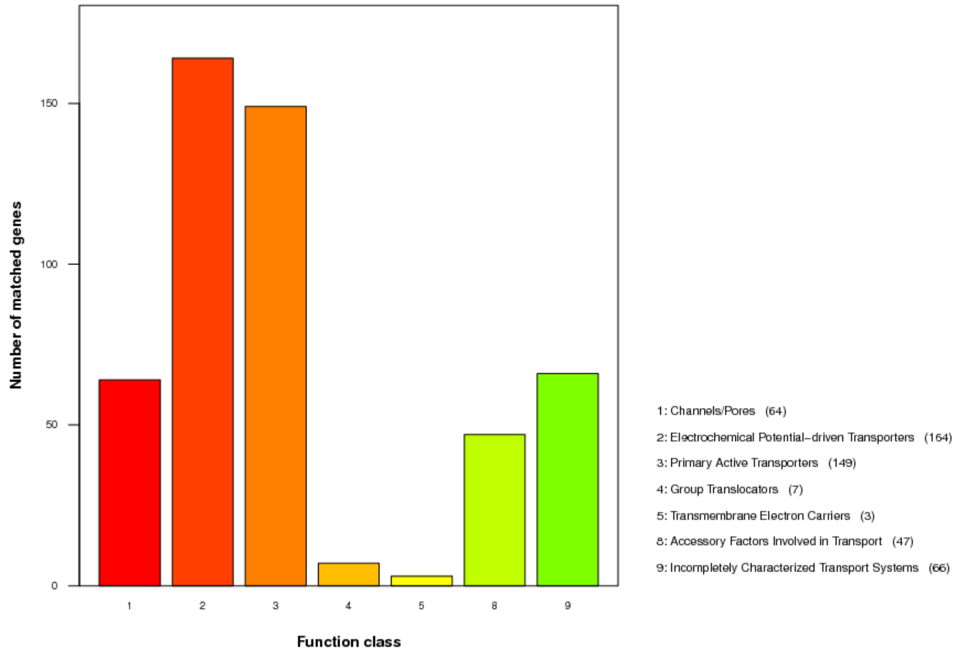

Figure S3. Functional classification diagram of TCDB in (A) M3 and (B) M34 gene annotations.

A

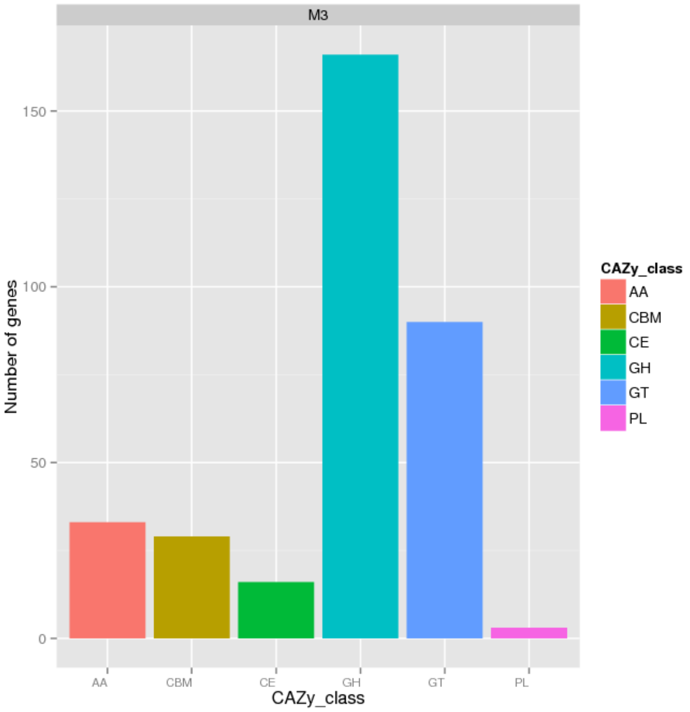

B

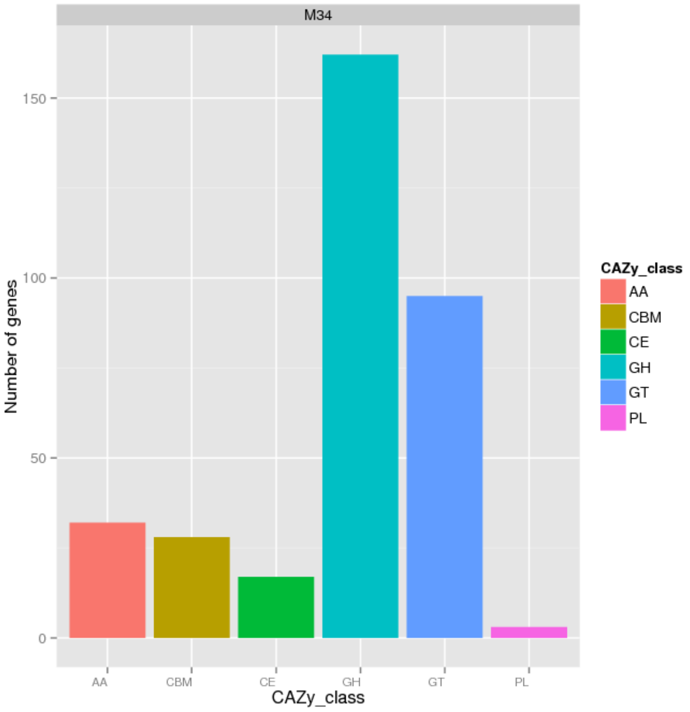

Figure S4. Functional classification diagram of CAZy in (A) M3 and (B) M34 gene annotations. AA, auxiliary activity; CBM, carbohydrate related modules; CE, carbohydrate esterase; GH, glycoside hydrolases; GT, glycosyltransferases; PL, polysaccharide lyases.

A

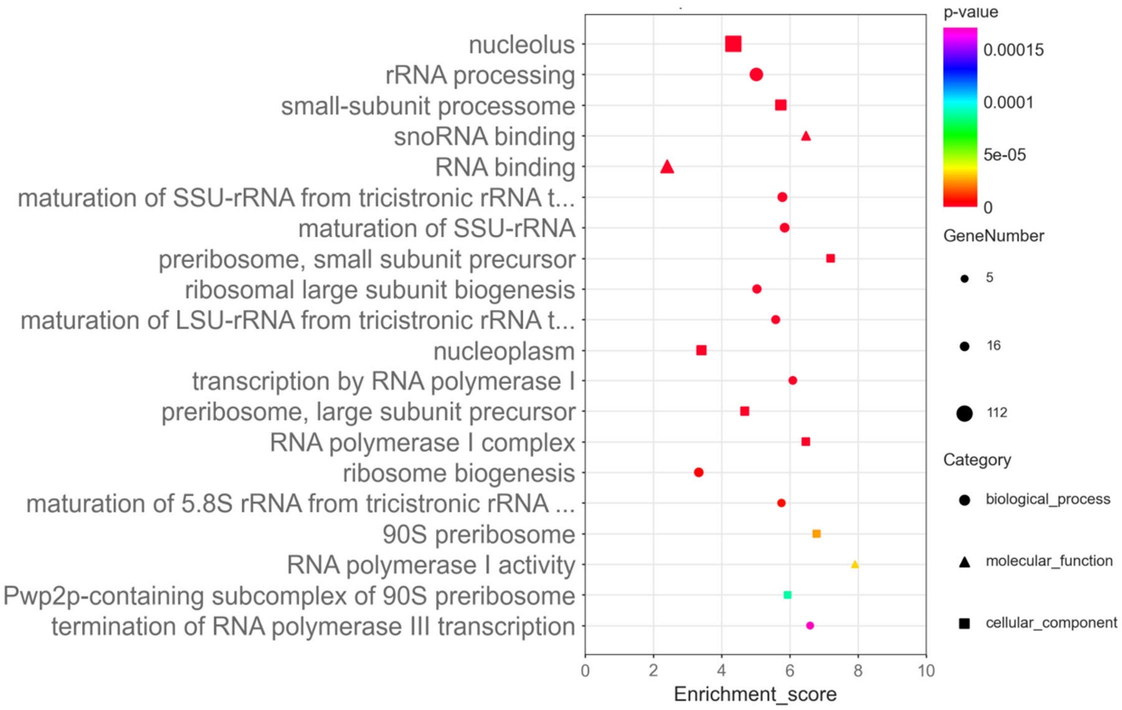

B

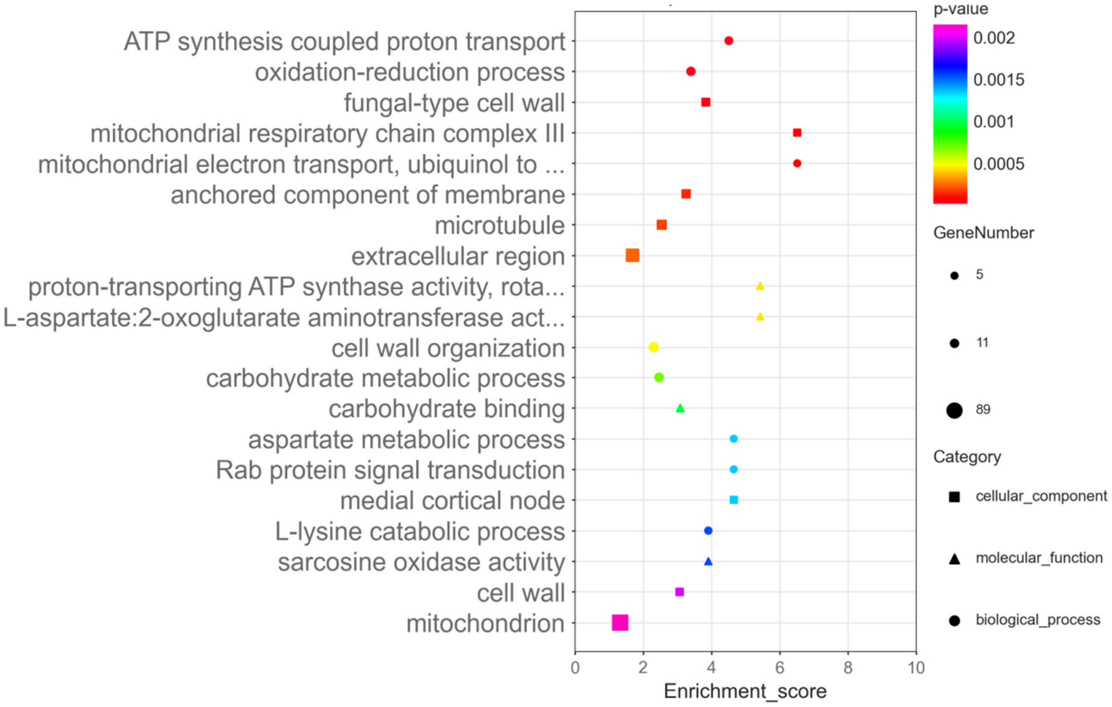

Figure S5. Functional classification of DEGs in strains M3 and M34. Scatter plot of the top 20 GO terms enriched in the downregulated (A) and the upregulated (B) DEGs in M34. Color indicates the significance level of enrichment.

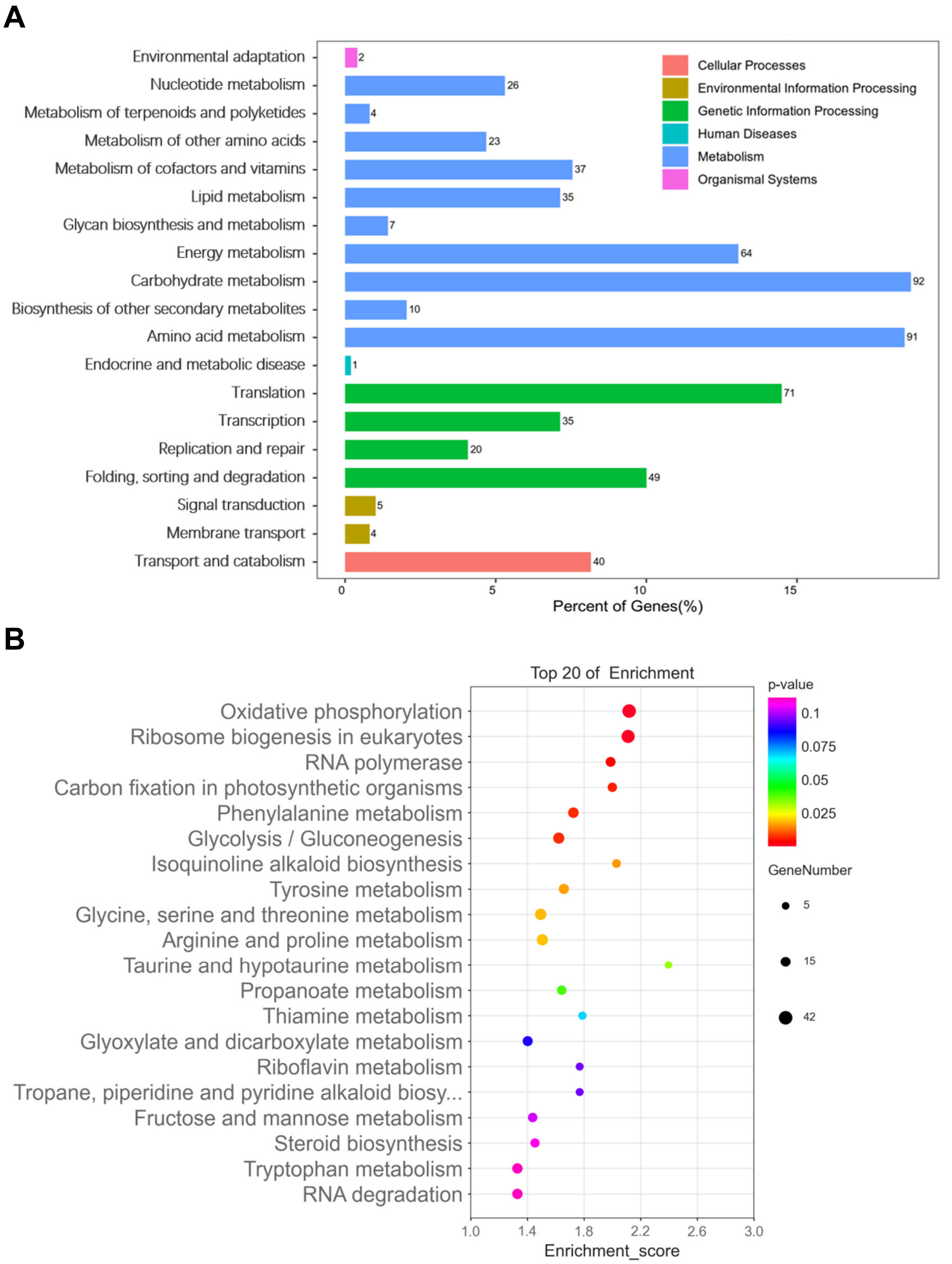

Figure S6. KEGG enrichment analysis of DEGs in *M. purpureus* strains M34 vs. M3. (A) KEGG enrichment pathways of DEGs. The Y-axis indicates the categories and subcategories of KEGG pathways, and the X-axis represents the percent of DEGs. (B) The top 20 pathways in the KEGG enrichment analysis of DEGs.

log2FC	Gene_id	NR annotation
-2.31	gene_5068	hypothetical protein MPDQ_000611
-2.27	gene_6905	hypothetical protein MPDQ_001107
-2.11	gene_8412	hypothetical protein MPDQ_001407
-2.06	gene_1955	hypothetical protein MPDQ_007923
-1.89	gene_5931	hypothetical protein MPDQ_002360
-1.85	gene_2826	hypothetical protein MPDQ_001231
-1.85	gene_7646	hypothetical protein C8Q69DRAFT_200752
-1.64	gene_9417	C6 transcription factor Prf
-1.55	gene_4813	hypothetical protein MPDQ_003590
-1.54	gene_6515	Citrinin biosynthesis transcriptional activator ctnR
-1.50	gene_6474	lactose regulatory protein lac9 and GAL4-like protein
-1.47	gene_5378	hypothetical protein MPDQ_005879
-1.44	gene_9797	hypothetical protein MPDQ_004505
-1.44	gene_8163	hypothetical protein MPDQ_002308
-1.43	gene_6420	hypothetical protein MPDQ_002609
-1.41	gene_4114	hypothetical protein MPDQ_003200
-1.21	gene_9761	hypothetical protein MPDQ_006315
-1.21	gene_3806	hypothetical protein MPDQ_000529
-1.15	gene_5633	hypothetical protein MPDQ_002792
-1.12	gene_8286	Maltose acetyltransferase
-1.10	gene_7254	hypothetical protein MPDQ_006517
-1.06	gene_6821	hypothetical protein MPDQ_002192
-1.06	gene_1291	hypothetical protein MPDQ_004674
-1.02	gene_5381	hypothetical protein MPDQ_005876
1.07	gene_7696	hypothetical protein MPDQ_004428
1.15	gene_4499	hypothetical protein MPDQ_003340
1.21	gene_5192	hypothetical protein MPDQ_001872
1.22	gene_5910	hypothetical protein MPDQ_002536
1.26	gene_34	hypothetical protein MPDQ_003611
1.27	gene_3443	hypothetical protein MPDQ_003532
1.31	gene_279	hypothetical protein MPDQ_001437
1.34	gene_4821	hypothetical protein MPDQ_006775
1.39	gene_1768	hypothetical protein MPDQ_003432
1.46	gene_8318	hypothetical protein MPDQ_004191
1.53	gene_7123	hypothetical protein MPDQ_001327
1.54	gene_3339	hypothetical protein PENDEC_c010G05275
1.66	gene_1606	hypothetical protein ASPWEDRAFT_266719
1.76	gene_6863	hypothetical protein MPDQ_007795
1.77	gene_4483	hypothetical protein MPDQ_002920
1.85	gene_7681	hypothetical protein MPDQ_002394
2.63	gene_1900	hypothetical protein MPDQ_007360
3.90	gene_8030	hypothetical protein MPDQ_000984

Figure S7. Expression fold changes (log_2FC) of selected DEGs that encode Fungal Zn(2)-Cys(6) binuclear cluster domain proteins.
